# Acetovanillone augmented the cardioprotective effect of carvedilol against cadmium-induced heart injury via suppression of oxidative stress and inflammation signaling pathways

**DOI:** 10.1038/s41598-023-31231-5

**Published:** 2023-03-31

**Authors:** Emad H. M. Hassanein, Adel G. Bakr, Ehab A. M. El-Shoura, Lamiaa Khalaf Ahmed, Fares E. M. Ali

**Affiliations:** 1 Department of Pharmacology and Toxicology, Faculty of Pharmacy, Al-Azhar University, Assiut Branch, Assiut, 71524 Egypt; 2 Biochemistry and Molecular Biology Department, Faculty of Pharmacy (Girls), Al-Azhar University, Assiut, Egypt; 3 Present Address: Department of Pharmacology and Toxicology, Faculty of Pharmacy, Al-Azhar University, Assiut Branch, Assiut, 71524 Egypt

**Keywords:** Pharmacology, Biochemistry, Chemical biology, Cardiology

## Abstract

Cardiac toxicity is a public health issue that can be caused by both environmental and occupational exposures. The current study aimed to investigate the effectiveness of carvedilol (CV), Acetovanillone (ACET), and their combination for ameliorating cadmium (Cd)-induced oxidative stress, inflammation, and necroptosis. Rats were assigned to; the normal group, Cd group (2 mg/kg; i.p., single dose), and the other three groups received orally CV (10 mg/kg), ACET (25 mg/kg), and CV plus ACET, respectively and a single dose of Cd. Oral administration of CV, ACET, and their combination significantly dampens cardiac oxidative injury by increasing antioxidants GSH and SOD levels, while it decreases MDA and NADPH oxidase levels mediated by decreasing cardiac abundance of Nrf2, HO-1, and SIRT1 and downregulating KEAP-1 and FOXO-3 levels. Also, they significantly attenuated inflammatory response as indicated by reducing MPO and NOx as well as proinflammatory cytokines TNF-α and IL-6 mediated by downregulating TLR4, iNOS, and NF-κB proteins expression as well as IκB upregulation. Moreover, they potently counteracted cardiac necroptosis by downregulating RIPK1, RIPK3, MLKL, and caspase-8 proteins expression. Of note, the combination of CV and ACET have marked protection that exceeded each drug alone. Conclusively, CV ad ACET potently mitigated Cd-induced cardiac intoxication by regulating NADPH oxidase, KEAP-1/Nrf2/HO-1, SIRT1/FOXO-3, TLR4/NF-κB/iNOS, and RIPK1/RIPK3/MLKL signals.

## Introduction

Heavy metal toxicity is a public health issue that can be caused by both environmental and occupational exposures. It endangers the environment and public health due to the widespread presence of metals in the earth's crust, and it can result in significant multiple organ damage and death after exposure to acute or chronic phase^1^. Like other toxic heavy metals, cadmium (Cd) is a very hazardous environmental pollutant that has been shown to cause toxicity in a variety of organs and cells^2,3^. Significantly, previous investigations have shown that Cd poisoning causes biochemical and pathological alterations in the heart, which eventually leads to cardiac dysfunctions in humans^4,5^ and animal models^6^. Cd causes cardiac damage by generating reactive oxygen species (ROS)^7,8^. Interestingly, the nuclear factor erythroid 2-related factor 2 (Nrf2)/antioxidant response element (ARE) signal is a defensive mechanism in response to oxidative and chemical stress by promoting the expression of antioxidants^9^. Under oxidative or chemical stress, kelch-like ECH-associated protein-1 (KEAP-1), the inhibitor of Nrf2, dissociates from Nrf2 and leads to its activation, and mediates cardiac protection^10,11^.

Toll-like receptors (TLRs) are pattern recognition receptors, which are a crucial component of the innate immune system^12^. Importantly, TLR4 mediates the inflammatory response in the heart^13,14^. Notably, Cd toxicity has a significant influence on TLRs and their downstream signaling process, and previous studies have shown that Cd activates the TLR gene in human epithelial cells via a stimulated TLR4-mediated signaling pathway^15,16^. Significantly, the TLR4 signal has been shown in multiple studies to govern the production of proinflammatory molecules and promote the inflammatory response in cardiac tissues, which is the main cause of myocardial tissue injury by activating nuclear factor-*kappa* B (NF-κB)^17,18^. Also, necroptosis, a type of programmed necrosis, is carried out via the activation of receptor-interacting protein kinase 3 (RIPK3) and its downstream substrate mixed lineage kinase-like domain (MLKL), which are both mediated by death receptors and play a crucial role in inflammation and cell death. The importance of necroptosis in heart diseases has been strongly supported by recent investigations^19^.

Interestingly, carvedilol (CV) is commonly used to treat different heart diseases. It also aids in preventing oxygen radicals from causing damage to nitric oxide-mediated coronary artery dilation^20^. CV has been shown to have potent cardioprotection mediated by antioxidative, anti-antiapoptotic properties^21,22^. Acetovanillone (ACET) is a natural phytochemical obtained from *Apocynum cannabinum* and *Picrorhiza kurroa*. ACET is believed to act as an antioxidant because it prevents the activity of the ROS-producing enzyme nicotinamide adenine nucleotide phosphate oxidase (NADPH), such as superoxide anion (O_2_^−^), and thus may be useful in the treatment of a variety of illnesses that evoked by an elevated inflammatory response. Besides its antioxidant, it also possesses anti-inflammatory effects in a variety of in vitro and animal models^23^. Therefore, based on the above evidence, we hypothesize that CV, ACET, or their combination could attenuate Cd-induced oxidative stress, inflammation, and necroptosis. As a result, we want to look into whether CV and ACET can protect rats from Cd-induced cardiac damage or not.

## Materials and methods

### Chemicals, primers, and antibodies

Carvedilol was purchased from GNP company (6th of October, Giza, Egypt). Cadmium chloride (CdCl_2_) and ACET were purchased from Sigma Aldrich (St. Louis, MO, USA). Rat tumor necrosis factor-alpha (TNF-α; CAT# E-EL-R2856), troponin-I (CAT# E-CL-R0721), and (IL-6; CAT# E-EL-R0015) ELISA kits were purchased from ELABSCIENCE (Wuhan, China). Primers for Sirtuin 1 (SIRT1), Forkhead box O3 (FOXO3), KEAP-1, Nrf2, and Glyceraldehyde 3-phosphate dehydrogenase (GAPDH) genes were synthesized by Vivantis Technologies (Malaysia). Anti-RIPK1 (CAT# PA5-20811), anti-RIPK3 (CAT# PA5-19956), anti-MLKL (CAT# PA5-115578), and anti-caspase-8 (CAT# PA5-20118) were purchased from ThermoFisher Scientific (USA). Anti-iNOS (CAT# sc-7271), IκB (CAT# sc-1643), and anti-TLR4 (CAT# sc-293072) were purchased from Santa Cruz (USA). Anti-NF-κB (CAT# abx012874) was purchased from Abbexa (Germany). Anti-NADPH oxidase (CAT# E-AB-70215) and anti-β-actin (CAT# E-AB-20031) were purchased from ELABSCIENCE (China). Anti-Nrf2 (CAT# YPA1865) and anti-HO-1 (CAT# YPA1919) were purchased from Biospes (China). ACET and CV dissolved in 0.5% carboxymethyl cellulose (CMC).

### Animal source and handling

Forty healthy male Wistar rats, averaging 12–14 weeks old, weighing between 180 and 200 g, were used in our study. Rats were purchased from the central animal house, Faculty of Medicine, Assiut University (Assiut, Egypt). A 12-h light/12-h dark cycle was used to keep the rats. Standard rodent food (not less than 19% proteins, 6% fibers, 3.5% fats, and 6.5% ash) and water are freely available to the animals. The rats were adapted to housing conditions for about seven days before the experimental design.

### Experimental design and samples collection

Male Wistar rats (n = 40), were randomly allotted into five groups, named control, Cd, ACET-Cd, CV-Cd, and ACET + CV-Cd groups, as follows:

- Group 1: (Served as the normal control group).
- Group 2: (To induce cardiotoxicity, the Cd-control group was injected with CdCl_2_ (2 mg/kg, i.p.) on day 5)^24^.
- Group 3: (CV, served as CV-treated group, rats administered with CV (10 mg/kg, p.o.) for 10 days and injected with CdCl_2_ (2 mg/kg, i.p.) on the 5th day of the experiment)^25^.
- Group 4: (ACET, served as ACET-treated group, rats administered with ACET (25 mg/kg, p.o.) for 10 days and injected with CdCl_2_ (2 mg/kg, i.p.) on the 5th day of the experiment)^26^.
- Group 5: (ACET plus CV, combination-treated group, rats were administered with ACET plus CV for 10 days and injected with CdCl_2_ (2 mg/kg, i.p.) on the 5th day of the experiment.

At the end of the experiment, all animals were anesthetized by i.p. administration of 100 mg/kg ketamine. In all groups, blood samples were collected retroorbitally for the determination of complete blood pictures (CBC). Serum was obtained after centrifugation at 4000 rpm for 10 min at 4 °C that was used for aspartate transaminase (AST), alkaline phosphatase (ALP), lactate dehydrogenase (LDH), creatine kinase myocardial band (CK-MB) and troponin-I evaluation. The anesthetized rats were sacrificed, and the cardiac tissue was cut into four sections. The first section was homogenized in phosphate buffer saline solution for the evaluation of oxidative stress biomarkers. The second section of cardiac tissue was mixed with lysis buffer for blotting analysis. The third section was mixed with RNAlater for real-time PCR estimation. The last section was fixed in a 10% formaldehyde solution for histopathological and immunohistochemical estimation.

### Assessment of heart function biomarkers

Serum CK-MB (CAT# 41254; Spinreact, Spain), ALP (CAT# 1001132; Spinreact, Spain), AST (CAT# 1001162; Spinreact, Spain), LDH (CAT# 1001260; Spinreact, Spain), and troponin-I were evaluated using commercial kits following the manufacturer’s directions.

### Histopathologic examination

Hematoxylin and eosin (H&E) staining and light microscopical examination of the heart paraffin blocks sectioned at a thickness of 4-µm was performed according to the method described by Bancroft and Steven^27^. A semiquantitative scoring system of histopathological findings was conducted according to the previously reported method^28^. The parameters of pathological changes observed were tabulated and graded using a scale of 0–4, where: (0): indicates the absence of the parameters in the samples or healthy myocardium. (1): indicating observed presence and distribution of the parameter up to 25% of the area examined (0–25%). (2): indicating observed presence and distribution of the parameter between 26 to 50% of the area examined (26–50%). (3): indicating observed presence and distribution of the parameter between 51 and 75% of the area examined (51–75%). (4): indicating observed presence and distribution of the parameter in more than 75% of the area examined (76–100%).

### Estimation of hematological indices

Hematological indices were estimated by conducting a complete blood count (CBC) using CellTac MEK-6510 (Japan) according to standard procedure^29^.

### Measurement of oxidative stress and antioxidant enzymes

The enzyme activity of myeloperoxidase (MPO) was estimated spectrophotometrically based on the peroxidation of dimethoxybenzidine by H_2_O_2_ in the presence of the enzyme according to the method described by Manktelow and Meyer^30^. Cardiac determination of reduced glutathione (GSH) and total nitrite end product (NO_2_^-^) levels were done according to the previously described methods by Sedlak and Lindsay^31^ and Montgomery and Dymock^32^, respectively. Colorimetric evaluation of superoxide dismutase (SOD) activity was performed according to the methods described by Marklund^33^, respectively. Lipid peroxidation was measured as malondialdehyde (MDA) according to the principle described earlier by Uchiyama and Mihara^34^.

### ELISA for proinflammatory cytokines

Cardiac TNF-α and IL-6 were measured by enzyme-linked immunosorbent assay according to the manufacturer's instructions. In brief, quantitative determination of TNF-α and IL-6 protein expression was performed by the addition of tissue samples containing TNF-α and IL-6 to microliter plates coated with purified rat TNF-α and IL-6 antibodies. Proteins of TNF-α and IL-6 combined with TNF-α and IL-6 antibodies labeled with avidin-HRP complex. This complex reacts with the TMB substrate to produce a blue color which converts to yellow color after sulfuric acid addition. The produced yellow color was measured at 450 nm using Readwell Strip, Elisa Reader, India.

### Immunohistochemistry

Using various grades of xylene and ethanol solutions, sections with a 4 µm thickness were placed on slides and deparaffinized. The slides were blocked with 1% BSA following antigen retrieval in citrate buffer (pH = 6). Primary antibodies for anti-iNOS (dilution 1:50), anti-NF-κB (dilution 1:50), and anti-TLR4 (dilution 1:50) antibodies were then incubated on the slides for an overnight period at 4 °C. A species-matched secondary antibody was added after washing to visually inspect the reaction for an hour at room temperature. The sections were counterstained with hematoxylin. The photos were taken with a digital camera attached to a light microscope. The area percentage of immunoreactivity was calculated using the ImageJ^®^ program.

### Quantitative real-time PCR

Total RNA was isolated from cardiac tissues using the Trizol reagent (Invitrogen, USA) according to the manufacturer’s standard instructions. Then, the obtained RNA was reverse-transcribed into cDNA following the manufacturer's instructions. Utilizing the sequence of a specific primer for each target gene, the generated cDNA was used for quantitative RT-PCR on the target genes. GAPDH was utilized as an internal control gene. Following the completion of the PCR amplification, the GAPDH Ct was subtracted from each sample's Ct to determine the expression of each targeted gene^35^. The primer sequences are registered in Table 1.

**Table 1:** Quantitative real-time PCR primers.

Gene	Forward primer	Reverse primer
Nrf2	ATTGCTGTCCATCTCTGTCAG	GCTATTTTCCATTCCCGAGTTAC
SIRT1	CGGTCTGTCAGCATCATCTTCC	CGCCTTATCCTCTAGTTCCTGTG
Keap1	TCAGCTAGAGGCGTACTGGA	TTCGGTTACCATCCTGCGAG
FOXO-3	GCCTCATCTCAAAGCTGGGT	AGTTCTGCTCCACGGGAAAG
GAPDH	TGCTGGTGCTGAGTATGTCG	TTGAGAGCAATGCCAGCC

### Western blotting

The protein levels of RIPK1, RIPK3, MLKL, and caspase-8 were assessed using western blot analysis. Protein concentrations were determined via Bradford protein assays^36^. Protein (50-μg) from each sample was loaded onto a 12.5% polyacrylamide gel. After electrophoresis, the proteins were transferred to a PVDF membrane using a semi-dry transfer method^37^. The membrane was incubated with primary antibodies (anti-RIPK1 (dilution 1:1000), anti-RIPK3 (dilution 1:1000), anti-MLKL (dilution 1:1000), anti-caspase-8 (dilution 1:1000), anti-NF-κB (dilution 1:2000), anti-NADPH oxidase (dilution 1:1000), anti-Nrf2 (dilution 1:500), anti-HO-1 (dilution 1:500), and anti-β-actin (dilution 1:3000)) overnight at 4 °C after being blocked with 5 percent defatted milk for 1 h. After 1 h at room temperature with an ALP-conjugated secondary antibody, the membrane was subjected to three washes with 0.1% TBS-Tween 20. The membrane was then subjected to detection using a BCIP/NPT detection kit after being rinsed five times with 0.1% TBS-Tween20. ImageJ^®^ software was used to quantify the bands. The protein levels were normalized by β-actin^38^.

### Statistical analysis

Statistical analysis was performed using GraphPad Prism software (GraphPad Software, version 7, USA). All values are presented as means ± SEM. Comparisons between groups were analyzed by a one-way ANOVA using Tukey’s post-hoc test at *P* < 0.05. Histopathological analysis was performed using Kruskal–Wallis followed by Tukey’s as post-hoc comparison test at *P* < 0.05.

### Ethical statement

This study was reviewed, and the protocol was approved by the Faculty of Pharmacy Animal Care and Use Committee of Al-Azhar University, Assiut Branch, Egypt (Approval number: ZA-AS/PH/4/C/2022). The current study adheres to the ARRIVE Guidelines for reporting in vivo experiments^67^. All experiments fulfill the guidelines of the National Institutes of Health guide for the care and use of laboratory animals (NIH Publications No. 8023, revised 1978)^68^ and the recommendations of Egypt's guide for the care and use of laboratory animals^69^.

## Results

### CV and ACET and their combination attenuate Cd-induced cardiac injury

**Figure 1:**
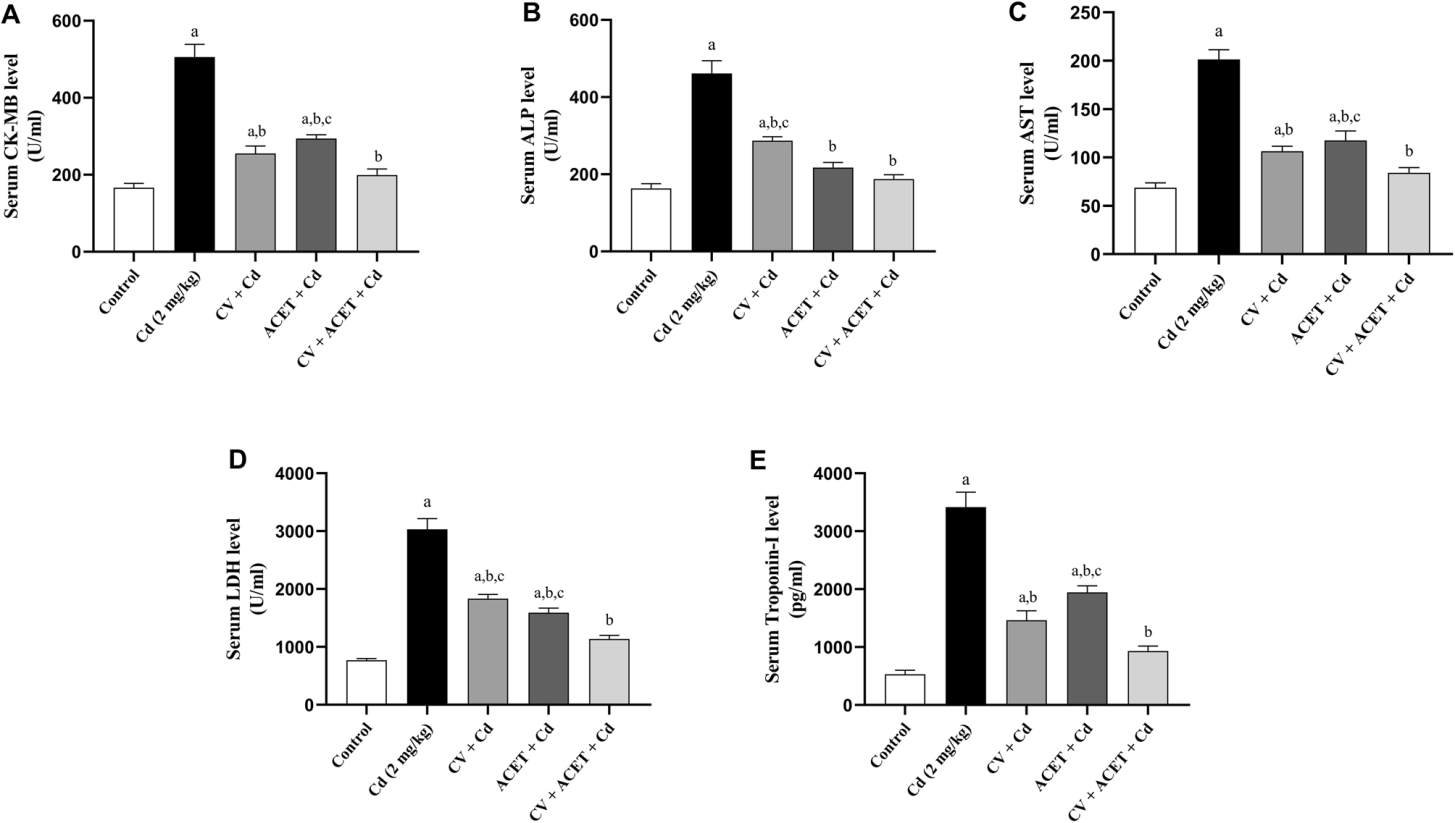
CV and ACET and their combination attenuate Cd-induced cardiac injury. To determine the cardioprotective effects of CV and ACET and their combinations against Cd-induced myocardial intoxication, serum CK-MB, ALP, AST, LDH, and troponin, were measured as indicating heart function biomarkers. Administration of CV, ACET, and their combination resulted in a remarkable decrease in serum CK-MB (**A**), ALP (**B**), AST (**C**), LDH (**D**), and troponin-I (**E**) levels with respect to that of the normal rats. Statistical analysis was conducted using one-way ANOVA followed by Tukey’s posthoc test. All values are presented as means ± SEM (n = 6). ^a^The significance Vs control group, ^b^the significance Vs Cd group, ^c^the significance Vs CV + ACET + Cd group at *P*-value < 0.05.

To determine the cardioprotective effects of CV and ACET and their combinations against Cd-induced myocardial intoxication, serum CK-MB, ALP, AST, LDH, and troponin-I were measured as indicating heart function biomarkers. As shown in Fig. 1, Cd injection resulted in a remarkable increase in serum CK-MB (Fig. 1A), ALP (Fig. 1B), AST (Fig. 1C), LDH (Fig. 1D), and troponin-I (Fig. 1E) levels by 204.3%, 182%, 192.5%, 294.4%, and 545% respectively, concerning that of the normal rats. On the contrary, these changes were counteracted in rats given either CV or ACET and their combinations. Of note, the combination of CV and ACET have marked protection exceeded each drug alone and exhibited values closer to the levels seen in the serum of normal control rats.

### CV and ACET and their combination attenuate Cd-induced cardiac histological abrasions

**Figure 2:**
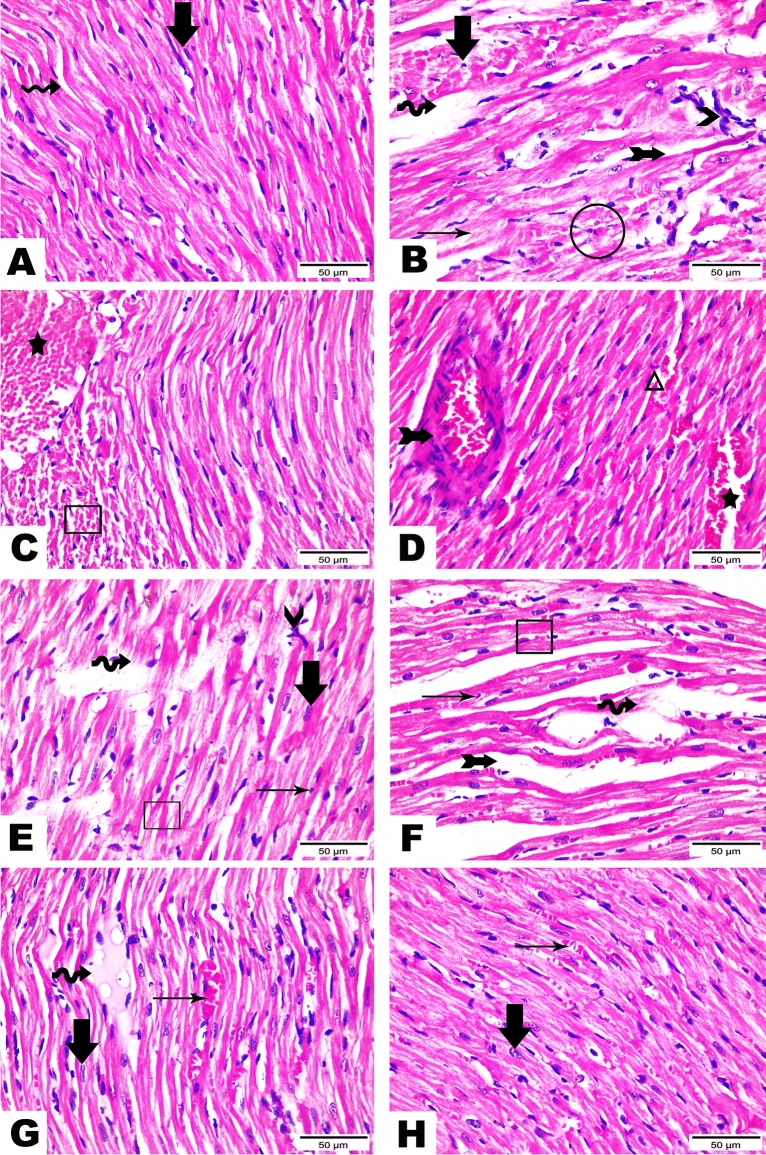
CV and ACET and their combination attenuate Cd-induced cardiac histological abrasions. Histological examinations notably confirmed the cardioprotective effect of CV and ACET and their combination. The normal heart of the normal control rats presents the characteristic histological structure of cardiomyocytes. They were marked as thin, branched, elongated cylinders, cross-striated along with large oval central nuclei (thick arrow). Also, constricted slit-like interstices were observed in between (wave arrow) (**A**). On the other hand, a rat’s heart injected with Cd highlights serious degenerative changes along myocardial fibers with loss of their normal architecture (two-head arrow), vacuolations (circle), and hyalinization of myofibrils (thin arrow), apoptotic myocytes (thick arrow), inflammatory cells infiltration (arrowhead), interstitial edema leading to dispersion between myofibrils (wave arrow), congested blood vessels (star) encircled by intermittent myofibrils (cube), and hemorrhage was dispersed in between myofibrils (triangle) (**B**–**D**). Rat’s heart was treated with Cd and CV revealing little improvement along heart tissue structure demonstrated by restoring most regularity of myofibrils but hemorrhage was still seen in few amounts (cube), cardiac myocytes appeared in two forms, normal (thick arrow) as well as apoptotic one (thin arrow). Hyalinized myofibrils (wave arrow), leucocytic cell infiltration (arrowhead), and interstitial edema leading to dispersion between myofibrils (two-head arrow) were also observed (**E**, **F**). Rat’s heart was injected with Cd and treated with ACET exhibiting a marked development evidenced by almost regular cardiac myocytes (thick arrow). Congestion of blood vessels, (wave arrow), mild hemorrhage (and little interstitial edema (thin arrow) are marked in between cardiac myofibrils (**G**). Interestingly, rat hearts treated with CV and ACET disclosed the best enhancement along tissue structure. Nearly all myocardial fibers restore normal assembly with large oval central nuclei of its myocytes (thick arrow) except some hemorrhage still presented between myofibrils (thin arrow) (**H**). Scale Bar = 50 μm.

Histological examinations notably confirmed the cardioprotective effect of CV and ACET and their combination. The normal heart of the normal control rats presents the characteristic histological structure of cardiomyocytes. They were marked as thin, branched, elongated cylinders, cross-striated along with large oval central nuclei (thick arrow). Also, constricted slit-like interstices were observed in between (wave arrow). On the other hand, a rat’s heart injected with Cd highlights serious degenerative changes along myocardial fibers with loss of their normal architecture (two-head arrow), vacuolations (circle), and hyalinization of myofibrils (thin arrow), apoptotic myocytes (thick arrow), inflammatory cells infiltration (arrowhead), interstitial edema leading to dispersion between myofibrils (wave arrow), congested blood vessels (star) encircled by intermittent myofibrils (cube), and hemorrhage was dispersed in between myofibrils (triangle). Rat’s heart was treated with Cd and CV revealing little improvement along heart tissue structure demonstrated by restoring most regularity of myofibrils but hemorrhage was still seen in few amounts (cube), cardiac myocytes appeared in two forms, normal (thick arrow) as well as apoptotic one (thin arrow). Hyalinized myofibrils (wave arrow), leucocytic cell infiltration (arrowhead), and interstitial edema leading to dispersion between myofibrils (two-head arrow) were also observed. Rat’s heart was injected with Cd and treated with ACET exhibiting a marked development evidenced by almost regular cardiac myocytes (thick arrow). Congestion of blood vessels, (wave arrow), mild hemorrhage (and little interstitial edema (thin arrow) are marked in between cardiac myofibrils. Interestingly, rat hearts treated with CV and ACET disclosed the best enhancement along tissue structure. Nearly all myocardial fibers restore normal assembly with large oval central nuclei of its myocytes (thick arrow) except some hemorrhage still presented between myofibrils (thin arrow) (Fig. 2).

**Figure 3:**
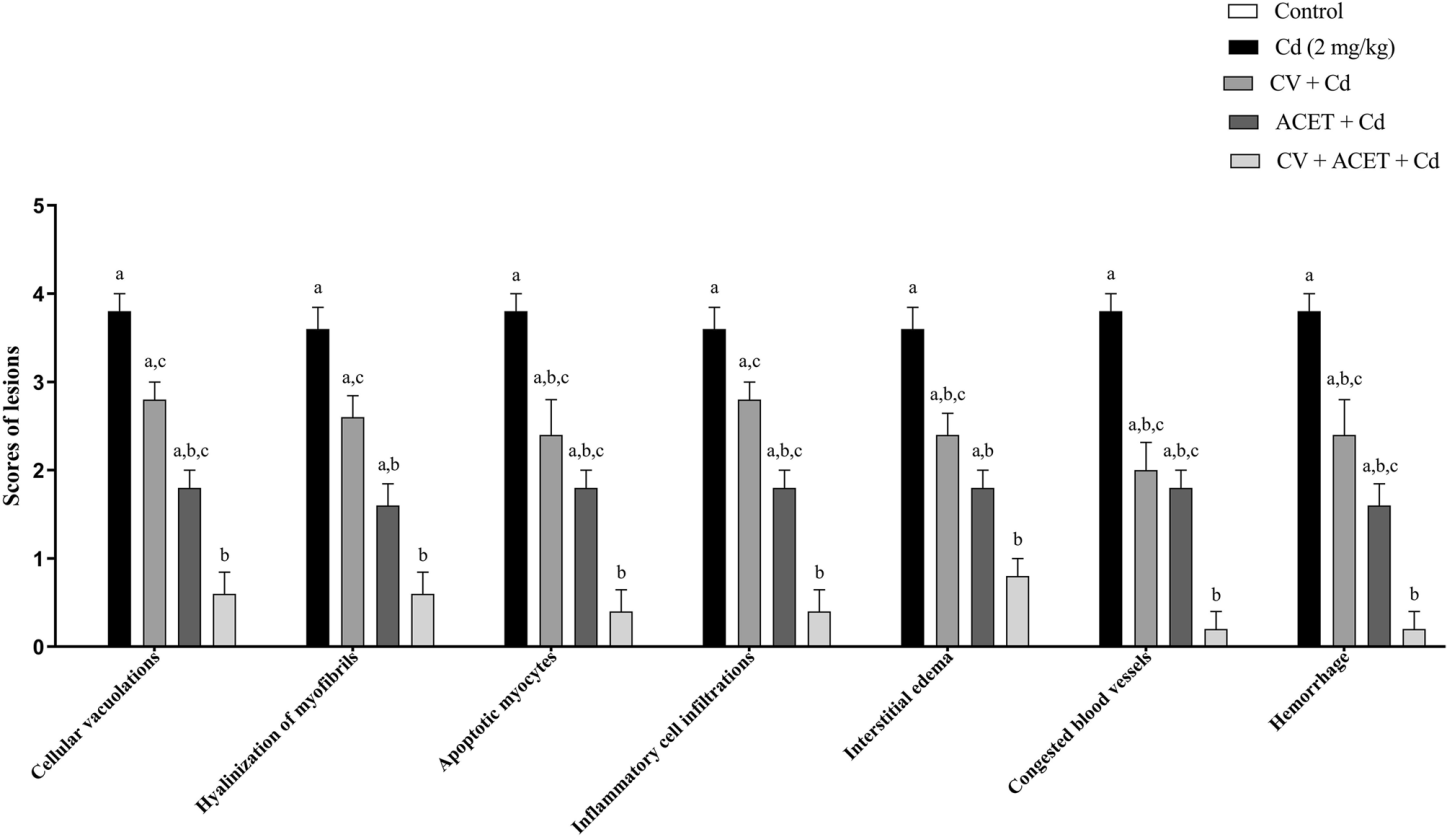
Semiquantitative scoring analysis for histopathological findings. Statistical analysis was conducted using the Kruskal–Wallis test followed by Tukey’s posthoc test. All values are presented as means ± SEM (n = 6). ^a^The significance Vs control group, ^b^the significance Vs Cd group, ^c^the significance Vs CV + ACET + Cd group at *P*-value < 0.05.

Furthermore, as shown in Fig. 3, quantitative analysis of histopathological findings showed that injection of Cd significantly increased all histopathological findings as indicated before compared to the control group (P < 0.05). However, most of these changes were significantly reversed by CV, ACET, and their combination treatment as compared to the Cd control group (P < 0.05).

### CV, ACET, and their combination fixed Cd-induced hematological abnormalities

**Figure 4:**
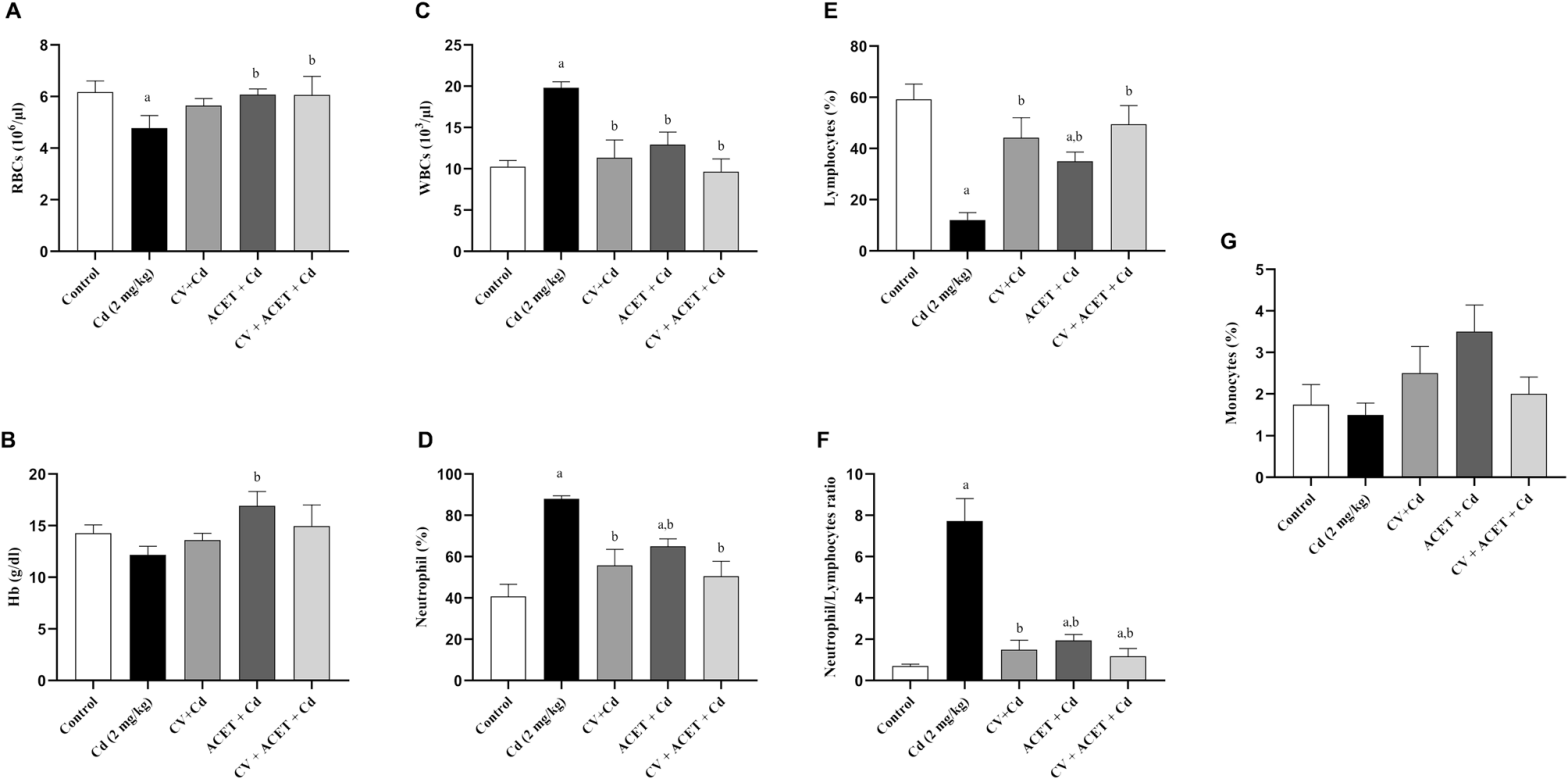
Effect of CV and ACET and their combination on hematological indices. RBCs (**A**), Hb (**B**), WBCs (**C**), Neutrophils (**D**), Lymphocytes (**E**), Neutrophil/Lymphocyte ratio (**F**), and Monocytes (**G**) were assessed. Statistical analysis was conducted using a one-way ANOVA test followed by Tukey’s posthoc test. All values are presented as means ± SEM (n = 6). ^a^The significance Vs control group, ^b^the significance Vs Cd group at *P*-value < 0.05.

The effect of CV, ACET, and their combinations on hematological indices was evaluated by CBC analysis. in the present study, Cd exposure caused a significant alteration in the total leukocyte and neutrophil count, and neutrophil/lymphocyte ratio. Notably, pretreatment with CV, ACET, and their combination markedly reverse Cd-induced hematological alteration with higher values in the combination therapy than in monotherapy (Fig. 4).

### CV and ACET and their combination attenuate Cd-induced cardiac oxidative injury

**Figure 5:**
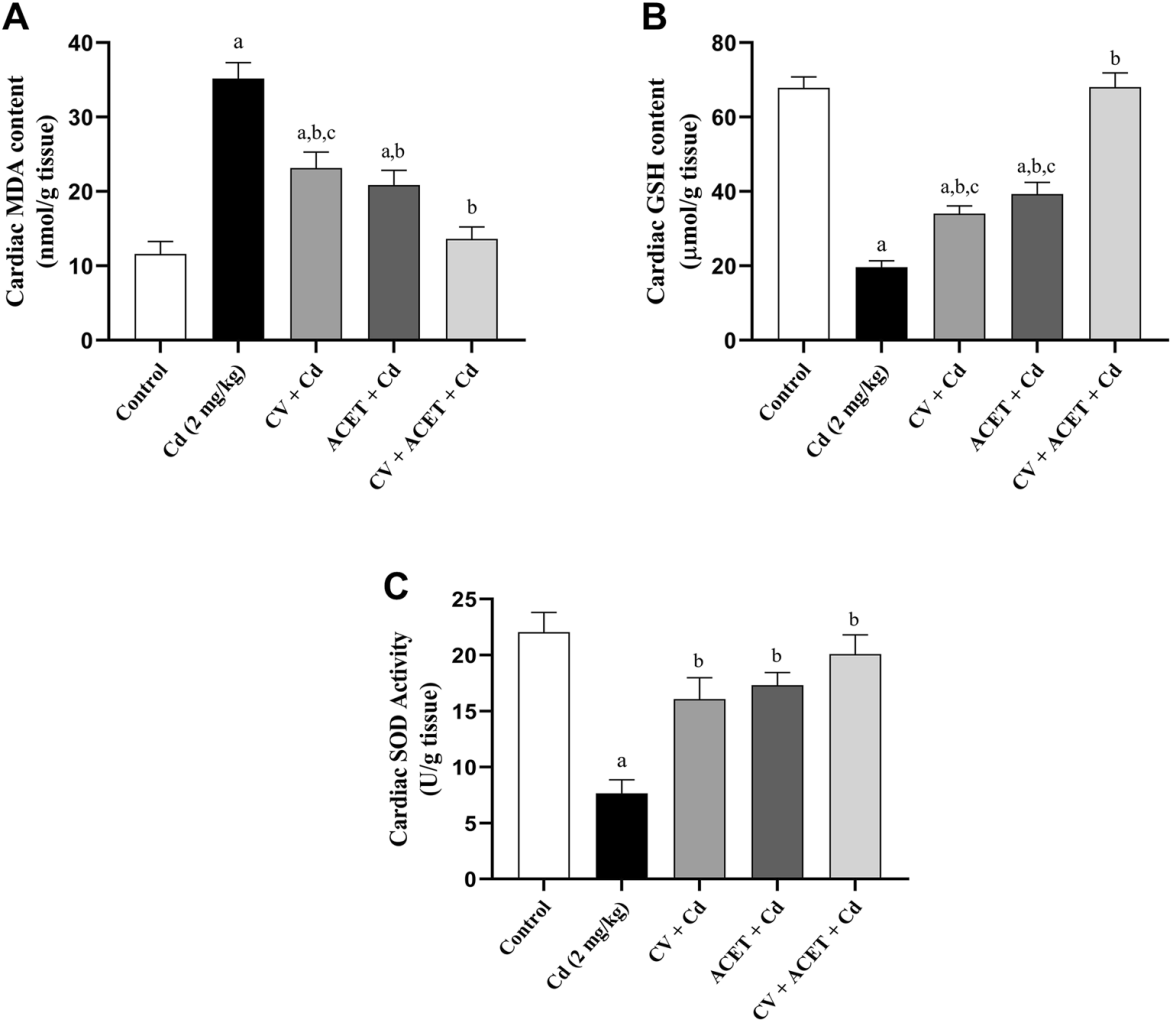
CV and ACET and their combination attenuate Cd-induced cardiac oxidative injury. Administration of CV, ACET, and their combination effectively increased GSH (**A**) and SOD (**B**) levels, while it decreased in MDA (**C**) content in the heart of rats. Statistical analysis was conducted using one-way ANOVA followed by Tukey’s posthoc test. All values are presented as means ± SEM (n = 6). ^a^The significance Vs control group, ^b^the significance Vs Cd group, ^c^the significance Vs CV + ACET + Cd group at *P*-value < 0.05.

To assess the effect of CV and ACET on Cd-induced cardiac oxidative damage, the non-enzymatic antioxidant GSH (Fig. 5A), antioxidant enzyme and SOD (Fig. 5B), as well as the lipid peroxidation biomarker MDA (Fig. 5C) were determined. In Cd control rats, cardiac GSH and SOD levels were significantly decreased by 71%, and 65.3% respectively, whereas the cardiac MDA content was significantly increased by 203.4% relative to normal control rats. Conversely, co-treatment of Cd with CV, ACET, and their combination effectively increased GSH levels by 73.4%, 100.1%, and 246.5% respectively, and SOD levels by 109.9%, 126.3%, and 162.4% respectively, while it decreased MDA content by 34.2%, 40.6%, and 61.3% respectively, in the heart of rats.

### CV and ACET and their combination modulate redox-sensitive signals in Cd-induced cardiac intoxication in rats

**Figure 6:**
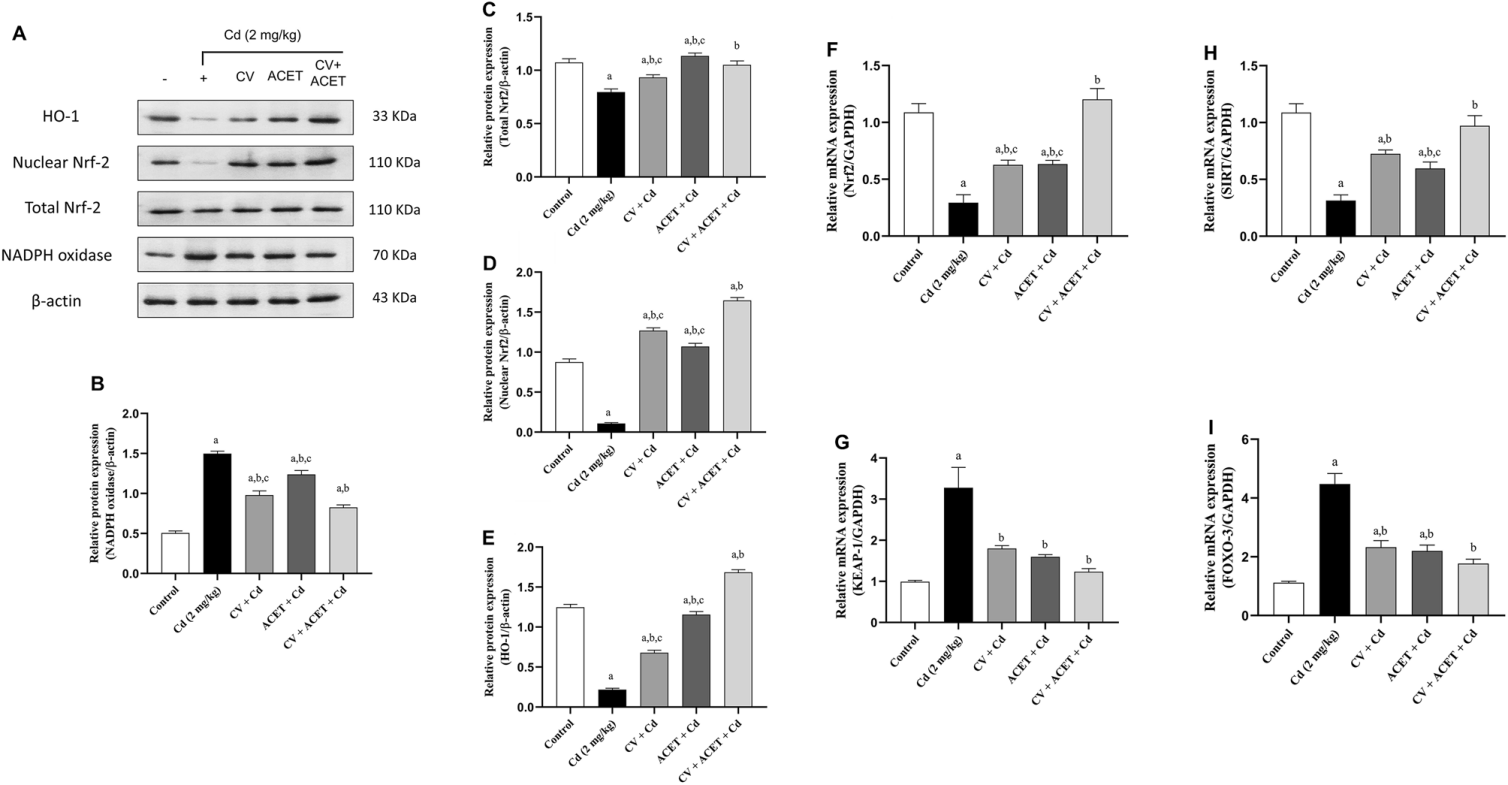
CV and ACET and their combination modulate molecular oxidant/antioxidant status in Cd-induced cardiac intoxication in rats. (**A**) Western blots of HO-1, Nrf2, NADPH oxidase, and β-actin protein expressions. The protein expression of NADPH oxidase/Nrf2/HO-1 was assessed using western blot analysis. Oral administration of CV, ACET, and their combination significantly downregulated NADPH oxidase (**B**) and upregulated total Nrf2 (**C**), nuclear Nrf2 (**D**), and HO-1 (**E**) expressions. The mRNA expression of redox-sensitive signals KEAP-1/Nrf2 and SIRT1/FOXO-3 were assessed using a qRT-PCR assay. Oral administration of CV, ACET, and their combination significantly increased cardiac mRNA abundance of Nrf2 (**F**) and SIRT1 (**H**), and downregulated both KEAP-1 (**G**) and FOXO-3 (**I**) mRNA expressions effectively. Statistical analysis was conducted using one-way ANOVA followed by Tukey’s posthoc test. All values are presented as means ± SEM (n = 4). ^a^The significance Vs control group, ^b^the significance Vs Cd group, ^c^the significance Vs CV + ACET + Cd group at *P*-value < 0.05.

At the molecular level, the effect of CV and ACET and their combination on oxidant/antioxidant signals were assessed. Cardiac protein expression of NADPH oxidase was significantly upregulated (2.96 fold) after animals exposure to Cd as compared to normal control rats (Fig. 6B). This upregulation was concomitant with significant downregulation of both total and nuclear Nrf2 (Fig. 6C,D) by − 0.25 and − 0.88 folds respectively, and HO-1 (Fig. 6E) expressions by − 0.83 fold as compared to normal control rats. Interestingly, oral administration of CV, ACET, and their combination significantly decreased cardiac NADPH oxidase expression and increased total and nuclear Nrf2 and HO-1 expression as compared to the Cd control group (Fig. 6). Additionally, the mRNA expression of redox-sensitive signals KEAP-1/Nrf2 and SIRT1/FOXO-3 were assessed using a qRT-PCR assay. In this regard, when compared to normal rats, the heart of rats injected with Cd alone showed a significant down-regulation of Nrf2 (Fig. 6F) and SIRT1 (Fig. 6H) mRNA expression by − 0.73 and − 0.71 folds respectively, while the level of KEAP-1 (Fig. 6G) and FOXO-3 (Fig. 6I) significantly upregulated by 3.3 and 3.9 folds respectively. On the other side, compared to the Cd control group, oral administration of CV, ACET, and their combination significantly increased cardiac mRNA abundance of Nrf2 and SIRT1, and downregulated both KEAP-1 and FOXO-3 mRNA expressions effectively (Fig. 6). As compared to each drug alone, combination therapy exhibited the best results regarding all redox-sensitive pathways.

### CV and ACET and their combination suppress cardiac inflammation in Cd-intoxicated rats

**Figure 7:**
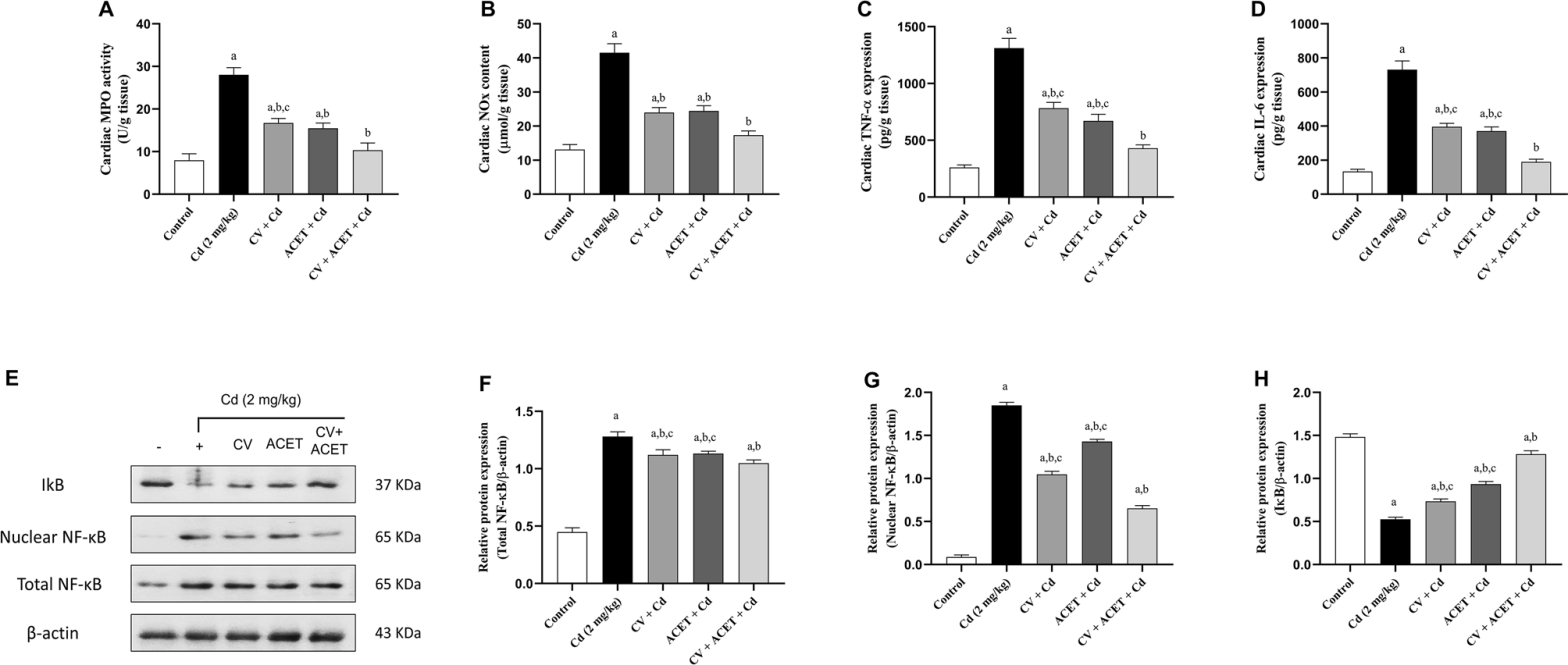
CV and ACET and their combination suppress cardiac inflammation in Cd-intoxicated rats. CV, ACET, and their combination significantly down-regulated MPO (**A**) and NOx (**B**) as well as proinflammatory cytokines TNF-α (**C**) and IL-6 (**D**) in the heart of rats. Oral CV, ACET, and their combination significantly down-regulated total NF-κB (**F**), nuclear NF-κB (**G**), and upregulated IκB (**H**) expression in the heart of rats. Statistical analysis was conducted using one-way ANOVA followed by Tukey’s posthoc test. All values are presented as means ± SEM (n = 3–6). ^a^The significance Vs control group, ^b^the significance Vs Cd group, ^c^the significance Vs CV + ACET + Cd group at *P*-value < 0.05.

Herein, Cd injection markedly upregulated MPO (Fig. 7A) and NOx (Fig. 7B) by 251.5% and 217.2% respectively, as well as proinflammatory cytokines TNF-α (Fig. 7C) and IL-6 (Fig. 7D) by 403.4% and 449.6% respectively, in the heart of rats compared to normal control rats. On the other side, CV, ACET, and their combination significantly attenuated these changes. Notably, the combination of CV and ACET brings them closer to the levels seen in normal control rats (Fig. 7). Additionally, we emphasized our results by investigating the protein expression of NF-κB (total and nuclear) and IκB in heart tissue using western blot assay. In parallel to other results, Cd injection caused a significant upregulation of NF-κB (total and nuclear) expression by 2.85 and 21.2 folds respectively, concomitant with downregulation of IκB cardiac protein expression by − 0.65-fold as compared to normal control rats (Fig. 7E–H). Interestingly, oral administration of CV, ACET, and their combinations significantly restored the balance between NF-κB and IκB as compared to the Cd control group (Fig. 7). Notably, the combination of CV and ACET exhibited a significant modulation of NF-κB/IκB compared to each drug alone.

### CV and ACET and their combination suppress iNOS/TLR4/NF-κB signal in Cd-intoxicated rats

**Figure 8:**
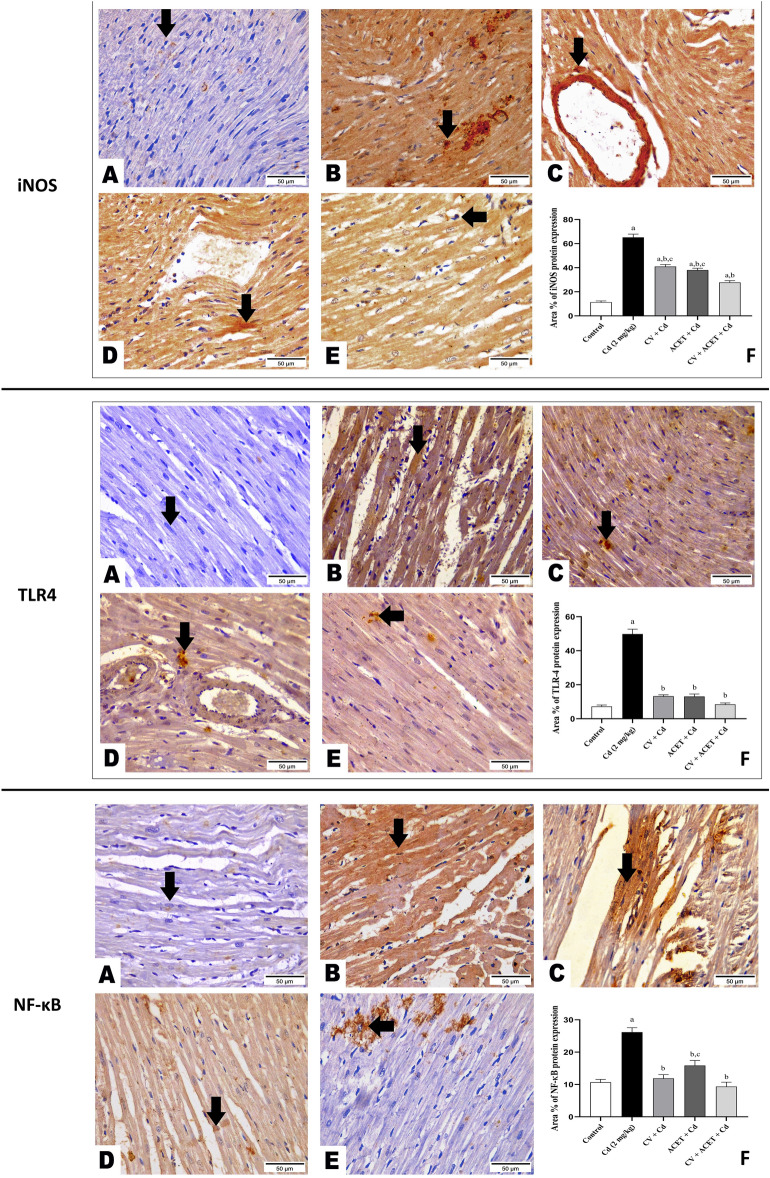
CV and ACET and their combination suppress iNOs/TLR4/NF-κB signal in Cd-intoxicated rats. We examined the effects on iNOS/TLR4/NF-κB signals using the IHC technique. Cd-intoxicated rats had a statistically significant increase in brown staining of iNOS, TLR4, and NF-κB compared to the normal rats. On the other side, treatment with CV, ACET, and their combination dramatically reduced the expression of iNOS, TLR4, and NF-κB proteins expression. (**A**) Control group, (**B**) Cd control group, (**C**) CV + Cd group, (**D**) ACET + Cd group, and (**E**) combination group. Statistical analysis was conducted using one-way ANOVA followed by Tukey’s posthoc test. All values are presented as means ± SEM (n = 4). ^a^The significance Vs control group, ^b^the significance Vs Cd group, ^c^the significance Vs CV + ACET + Cd group at *P*-value < 0.05.

Then, we aimed to explore more about the molecular mechanisms of the anti-inflammatory effects of CV and ACET on Cd-induced myocardial intoxication. Therefore, we examined their effects on iNOS/TLR4/NF-κB signals using the IHC technique. The results showed that Cd-intoxicated rats had a statistically significant increase in immunopositive expressions of iNOS (5.7-fold), TLR4 (6.8-fold), and NF-κB (2.6-fold) compared to the normal rats. Conversely, treatment with CV, ACET, and their combination dramatically reduced TLR4, iNOS, and NF-κB protein expressions as compared to the Cd control group (Fig. 8).

### CV and ACET suppress cardiac necroptosis in Cd-intoxicated rats

**Figure 9:**
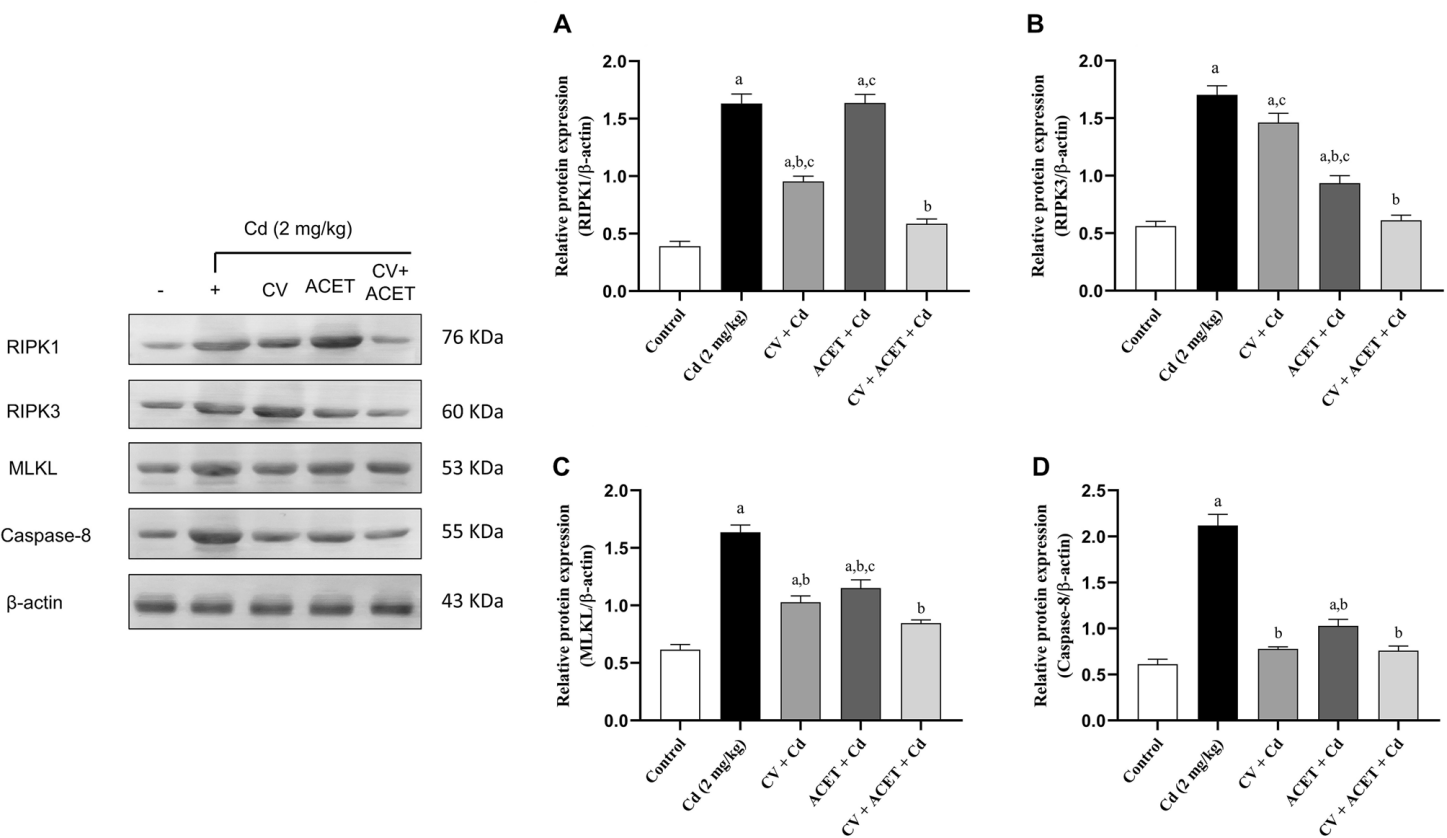
CV and ACET suppress cardiac necroptosis in Cd-intoxicated rats. We examined the expression levels of RIPK1, RIPK3, MLKL, and caspase-8 proteins using the western blot technique. CV, ACET, and their combination potently downregulated RIPK1 (**A**), RIPK3 (**B**), MLKL (**C**), and caspase-8 (**D**) protein expression. Statistical analysis was conducted using one-way ANOVA followed by Tukey’s posthoc test. All values are presented as means ± SEM (n = 3). ^a^The significance Vs control group, ^b^the significance Vs Cd group, ^c^the significance Vs CV + ACET + Cd group at *P*-value < 0.05.

Furthermore, we aimed to explore the role of necroptosis in Cd-induced myocardial intoxication. Therefore, we examined the expression levels of RIPK1, RIPK3, MLKL, and caspase-8 proteins using the W.B technique. Our data revealed that Cd-intoxicated rats had a statistically significant increase in RIPK1 (Fig. 9A), RIPK3 (Fig. 9B), MLKL (Fig. 9C), and caspase-8 (Fig. 9D) proteins by 4.2, 3.1, 2.7, and 3.5 folds respectively, compared to the normal rats. On the other side, treatment with CV, ACET, and their combination potently downregulated RIPK1, RIPK3, MLKL, and caspase-8 proteins expression.

## Discussion

International legislation has placed restrictions on Cd exposure. Individuals may be exposed to Cd at work or in the environment in a variety of ways, which could have public health repercussions. Ingestion of Cd-contaminated food and water is the primary source of exposure for nonsmokers^39^. In terms of the cardiovascular system, there is a growing body of epidemiological data suggesting Cd has a negative impact on heart health^40^. Cd deposition and accumulation in the heart and vascular tissue have also been found in humans^41^ and laboratory animals^42^. Cd accumulates in the body because of its lengthy half-life, which ranges from 10 to 40 years^43^. Epidemiological surveys also show a link between Cd and cardiovascular diseases^44,45^.

For the first time, the potential protective impact of CV, ACET, and their combinations on Cd-induced cardiac intoxication were revealed in this investigation. The serum levels of CK-MB, ALP, AST, LDH, and troponin were all significantly increased after a Cd injection. In contrast, when rats were given CV, ACET, or a combination of the two, these changes were reversed. On histological analysis, we found significant structural and functional changes in the Cd-control rats. Priya et al. (2017) revealed significant cardiac histological, functional, and biochemical changes in Cd-intoxicated rats^46^.

Several studies reported the significance of oxidative stress in the pathogenesis of cardiovascular diseases^47^. Cell membranes are the primary targets for Cd toxicity, followed by lipid peroxidation, protein oxidation, and thiol reduction^48^. The current study's findings show a rise in myocardial LPO levels following Cd administration, which could be due to an excess of free radical production and a reduction in antioxidant defense. In contrast, the administration of CV, ACET, and their combinations successfully decreased the levels of LPO and potently increased antioxidants in Cd-injected rats, indicating that CV, ACET, and their combinations had anti-lipid peroxidative and antioxidant actions.

Interestingly, the transcription factor Nrf2 has been found to regulate genes involved in cell signaling, transcription, and organ development. Significantly, Nrf2 is involved in cardiovascular disease and regulates the expression of antioxidant and detoxifying genes. The evidence suggests that Nrf2 protects the heart in experimental models of atherosclerosis, ischemia, heart failure, and others^9^. A growing body of research indicates that the transcription factor Nrf2 is essential for regulating the production of antioxidant genes, which ultimately have anti-inflammatory effects by suppressing NF-κB activation and cytokines production^49^. Moreover, SIRT1, a class III histone deacetylase that is NAD+-dependent, has been demonstrated to improve cell resistance and survival to stress. SIRT1 overexpression upregulates antioxidants, protecting the heart from oxidative stress^50,51^. Additionally, SIRT1 was reported to suppress NF-κB activation, a crucial controller of the proinflammatory signaling pathway^52^. The current study also showed that rats exposed to Cd had much lower levels of Nrf2, HO-1, and SIRT1 in the heart and significantly higher levels of NADPH oxidase, KEAP-1, and FOXO-3. In contrast, CV, ACET, and their combinations had antioxidant and anti-inflammatory effects that increased Nrf2 and SIRT1 expression levels. Our findings are consistent with earlier studies^53–56^. These data imply that CV and ACET's protection against Cd-induced myocardial oxidative stress may be due to KEAP-1/Nrf2/HO-1/SIRT1 upregulation concomitant with NADPH oxidase inhibition.

Increased levels of ROS may activate the first step in the signaling process that activates redox-sensitive NF-κB signaling^57,58^. Heart diseases have all been linked to increased iNOS expression. iNOS is a protein that is produced in the heart in response to inflammatory stimuli and produces NO. NO overproduction is cytotoxic and has been linked to cardiovascular disease. In addition, iNOS produces superoxide anion, which combines with NO to form the damaging oxidant peroxynitrite, resulting in cardiac oxidative injury^59,60^. Peroxynitrite is a powerful oxidant that causes cell necrosis or apoptosis. It is hazardous to cells due to DNA and protein changes and is one of the most potent oxidants in biological systems^61^. Simultaneously, several investigators discovered that NF-κB activation plays a key role in Cd-induced myocardial injury^62,63^. NF-κB inhibition improved Cd-induced heart damage, according to Ansari, et al.^64^ and Ge, et al.^65^. Here, Cd injection significantly increased MPO, NOx, and the inflammatory cytokines TNF-α and IL-6 in the rat heart mediated by TLR4/iNOs/NF-κB activation. On the other hand, these effects were greatly mitigated by CV, ACET, and their combination.

It has been suggested that necroptosis plays a significant role in the pathogenesis of various heart conditions^66^. Therefore, treating cardiovascular disorders may benefit from focusing on necroptosis signaling pathways. Consequently, we aimed to explore the role of necroptosis in Cd-induced myocardial intoxication as a novel possible mechanism of pathogenesis of Cd-induced cardiac intoxication. Our study showed that rats given Cd injections had considerably upregulated RIPK1, RIPK3, MLKL, and caspase-8. As a result, our research adds to the growing body of data indicating the RIPK1/RIPK3/MLKL signal is crucial to Cd-induced necrosis and sheds light on how Cd causes cardiac intoxication. Conversely, CV, ACET, and their combinations appear to prevent this signaling activation by suppressing cardiac necroptosis. It is noteworthy that our study is the first to shed light on CV and ACET anti-necrotic and anti-apoptotic functions in relation to the regulation of necroptosis signaling.

Future work is recommended to assess the effects of chronic exposure to Cd on the heart and its correlation with its accumulation in heart tissue to find out whether Cd tissue accumulation correlates with cardiac toxicity parameters (Supplementary information).

## Conclusions

In the present investigation, the administration of CV, ACET, and their combination significantly dampens cardiac oxidative injury by modulating NADPH oxidase, KEAP-1/Nrf2/HO-1, and SIRT1/FOXO-3. Also, they significantly attenuated inflammatory response by downregulating TLR4/iNOS/NF-κB signal. Moreover, they potently counteracted cardiac necroptosis by downregulating the RIPK1/RIPK3/MLKL signal. Of note, the combination of CV and ACET has marked protection that exceeded each drug alone. These promising effects were attributed to the potent antioxidative, anti-inflammatory, and anti-necroptotic activities of CV and ACET.

## Supplementary Information

Supplementary Information.

## Data Availability

The datasets used and/or analyzed during the current study are available from the corresponding authors upon reasonable request.
