# Supplementary material for: Acetovanillone augmented the cardioprotective effect of carvedilol against cadmium-induced heart injury via suppression of oxidative stress and inflammation signaling pathways
Source: Sci Rep. 2023 Mar 31;13:5278. doi: 10.1038/s41598-023-31231-5 (PMC10066216; doi:10.1038/s41598-023-31231-5)
Supplement: Supplementary file 1 — Supplementary Information. [file 41598_2023_31231_MOESM1_ESM.pdf]

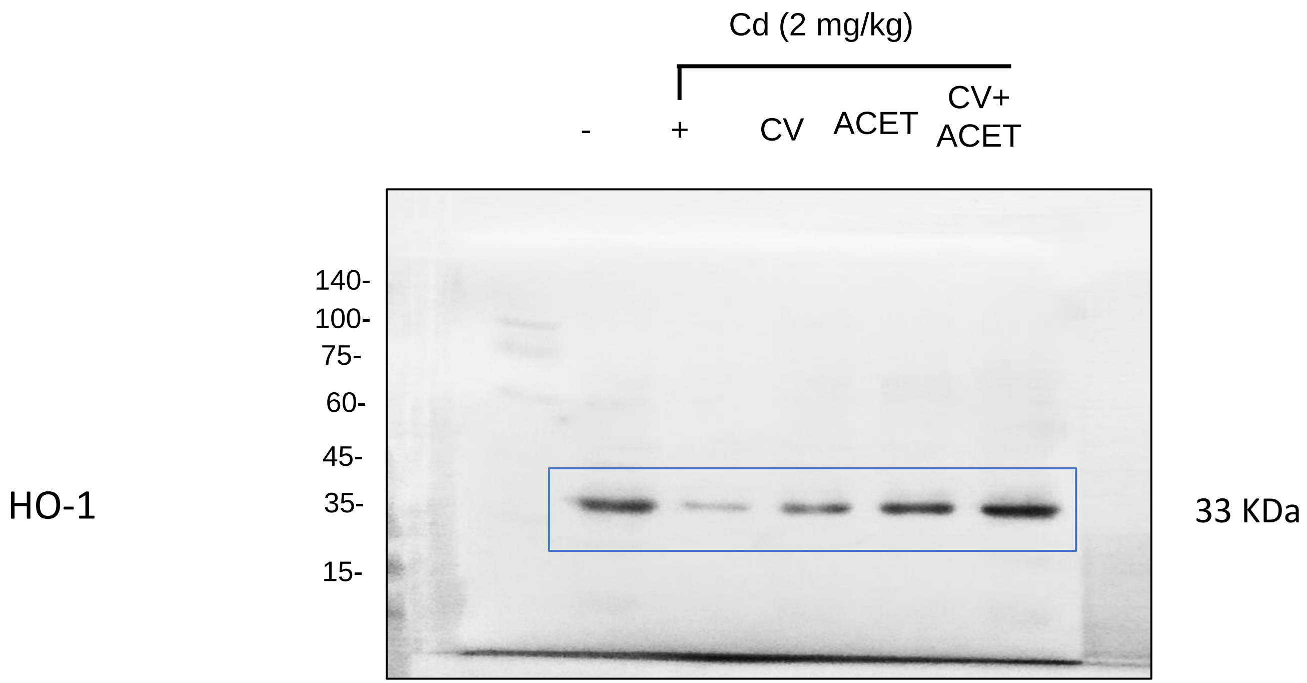

Cd (2 mg/kg)
-
+
CV
ACET
CV+ ACET
HO-1
140-
100-
75-
60-
45-
35-
15-
33 KDa

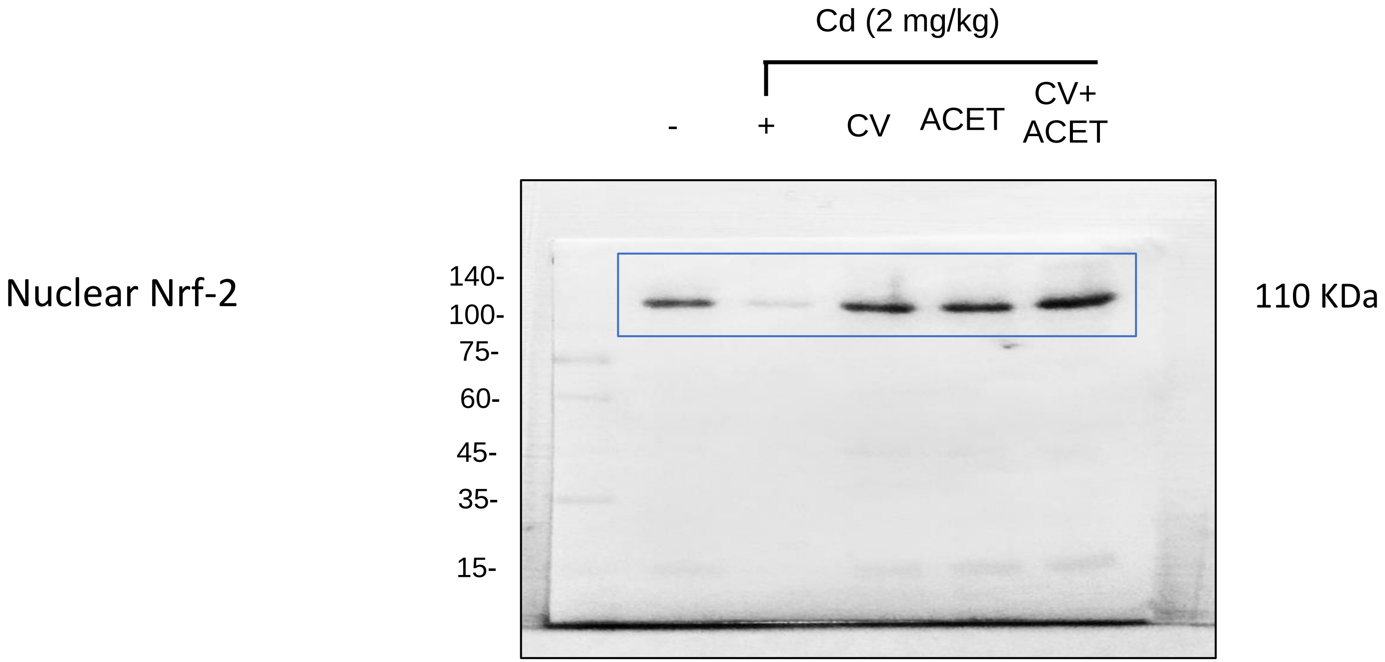

Cd (2 mg/kg)
-
+
CV
ACET
CV+ ACET
Nuclear Nrf-2
140-
100-
75-
60-
45-
35-
15-
110 KDa

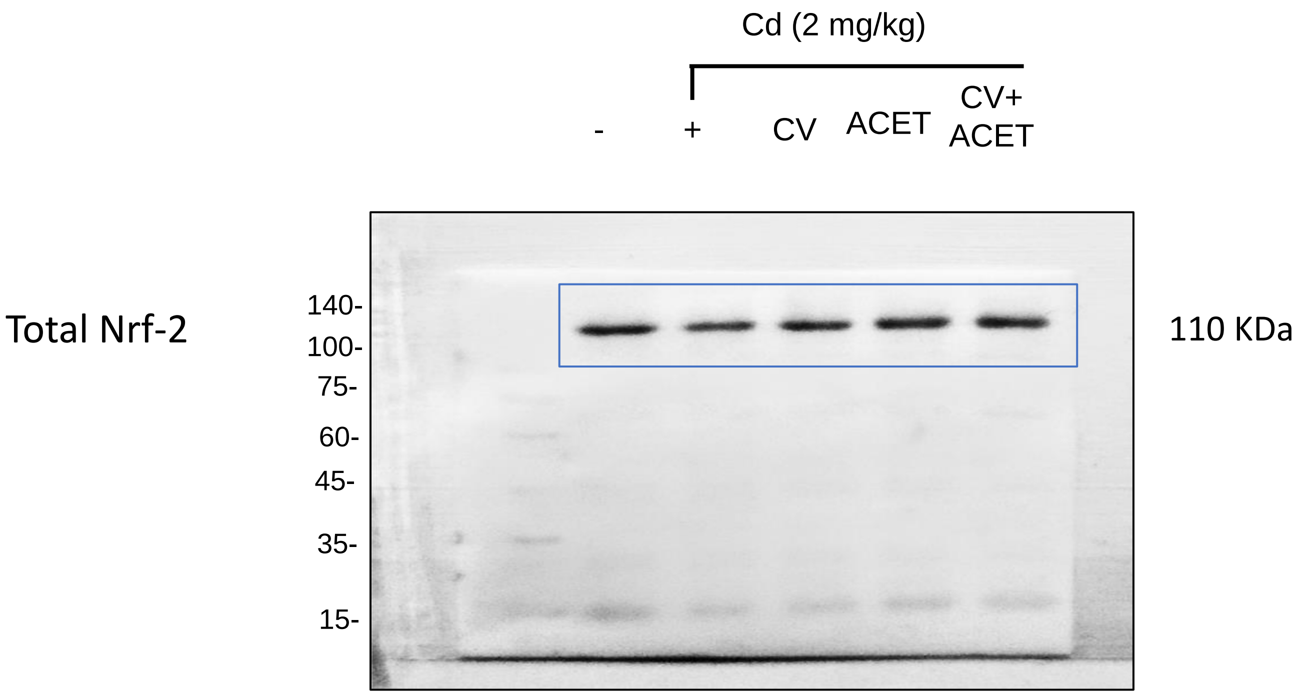

Cd (2 mg/kg)
-
+
CV
ACET
CV+ ACET
Total Nrf-2
140-
100-
75-
60-
45-
35-
15-
110 KDa

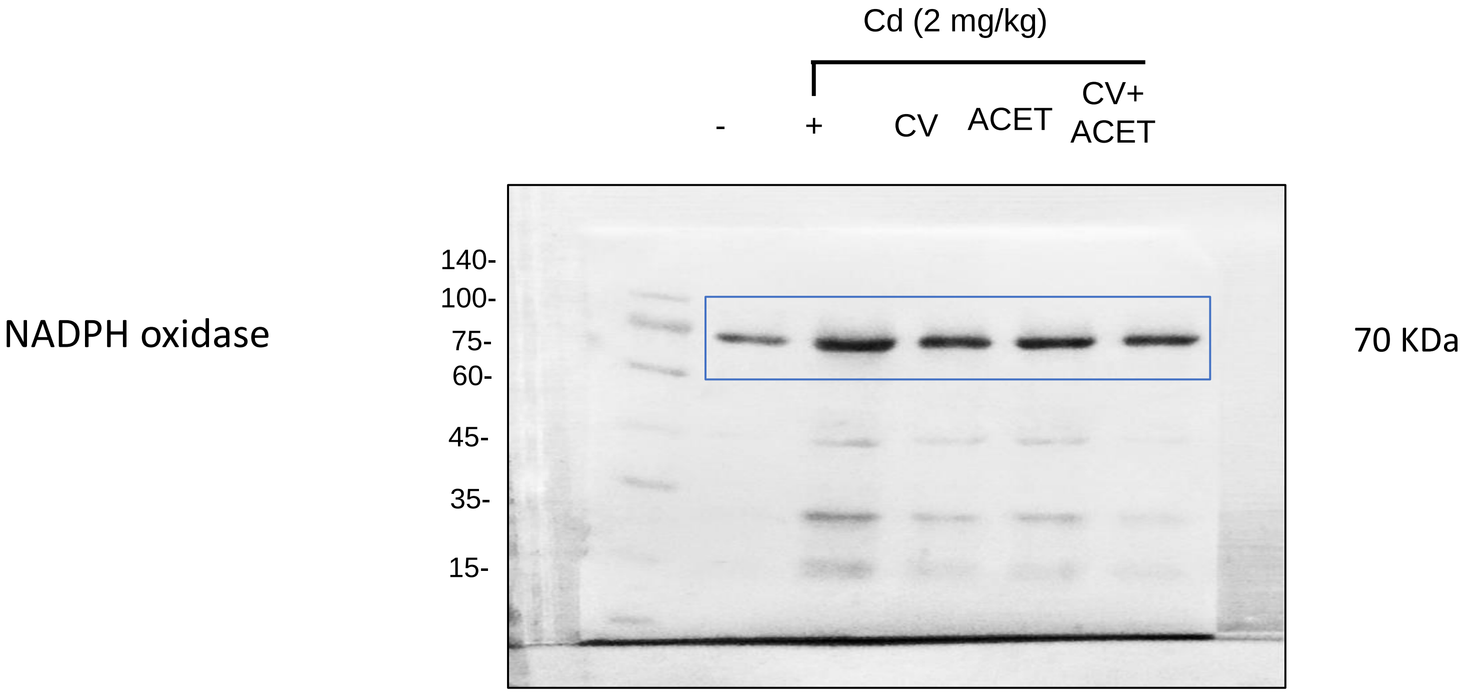
Cd (2 mg/kg)
-
+
CV
ACET
CV+ ACET
NADPH oxidase
140-
100-
75-
60-
45-
35-
15-
70 KDa

β-actin

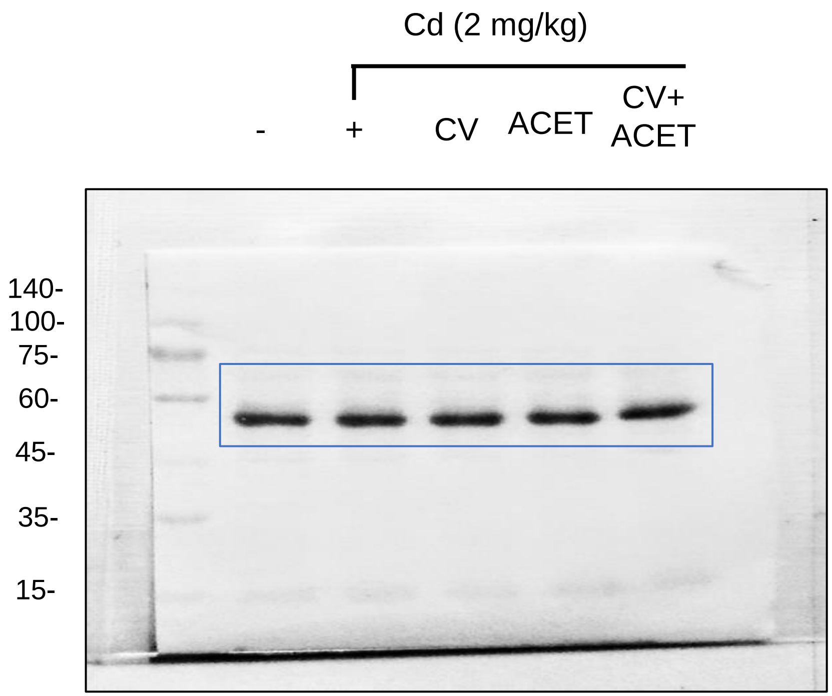

43 KDa

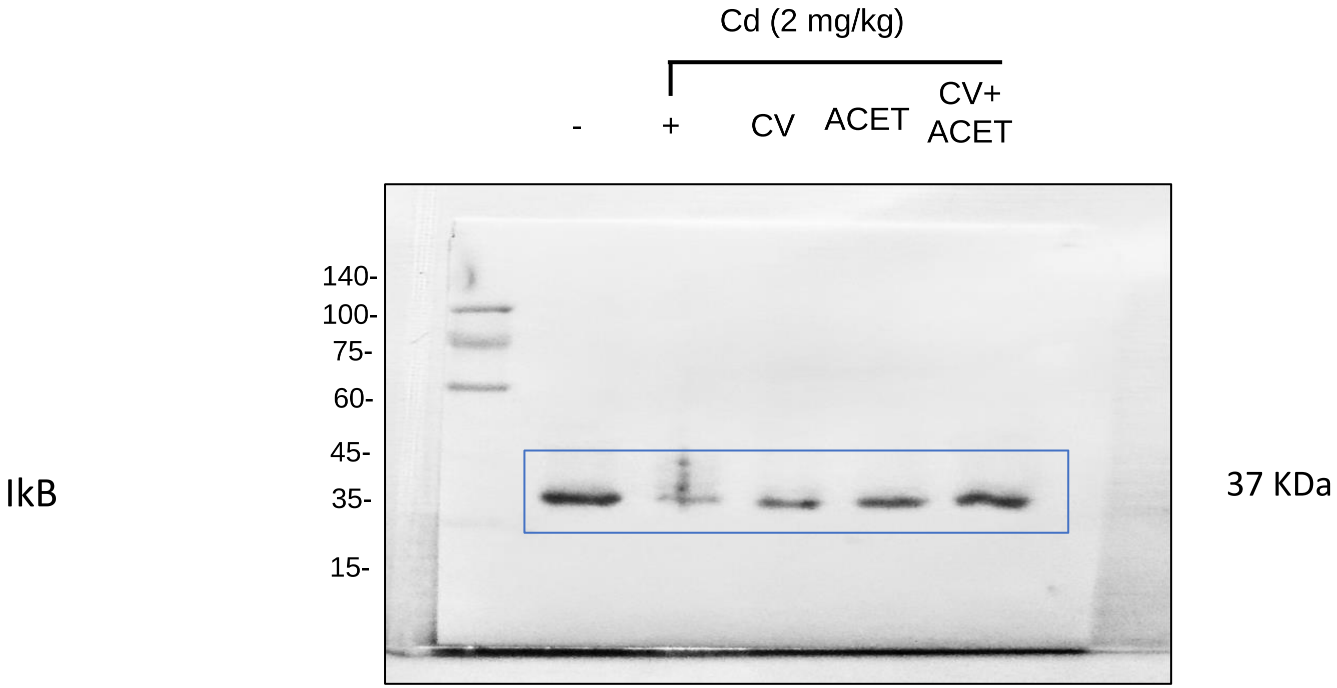
Cd (2 mg/kg)
-
+
CV
ACET
CV+
ACET
140-
100-
75-
60-
45-
35-
15-
IkB
37 KDa

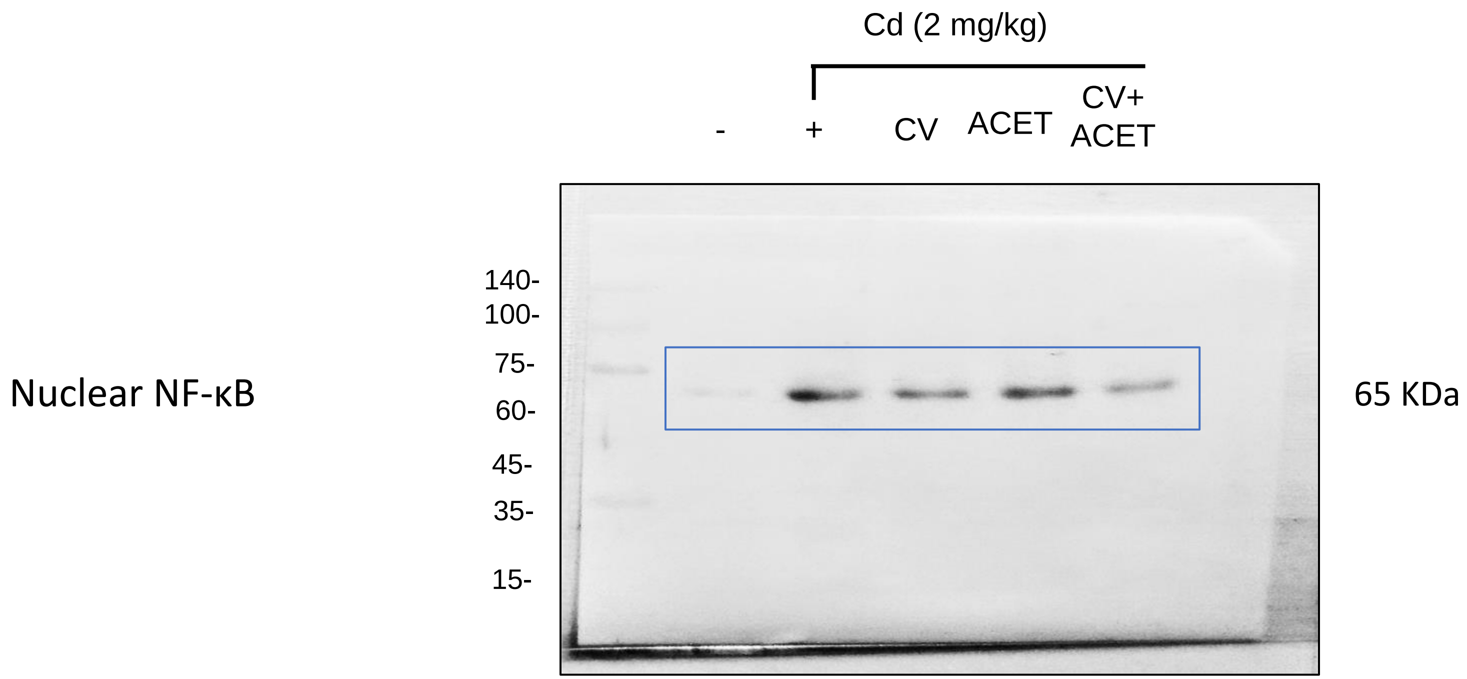
Cd (2 mg/kg)
-
+
CV
ACET
CV+ ACET
140-
100-
75-
60-
45-
35-
15-
Nuclear NF-κB
65 KDa

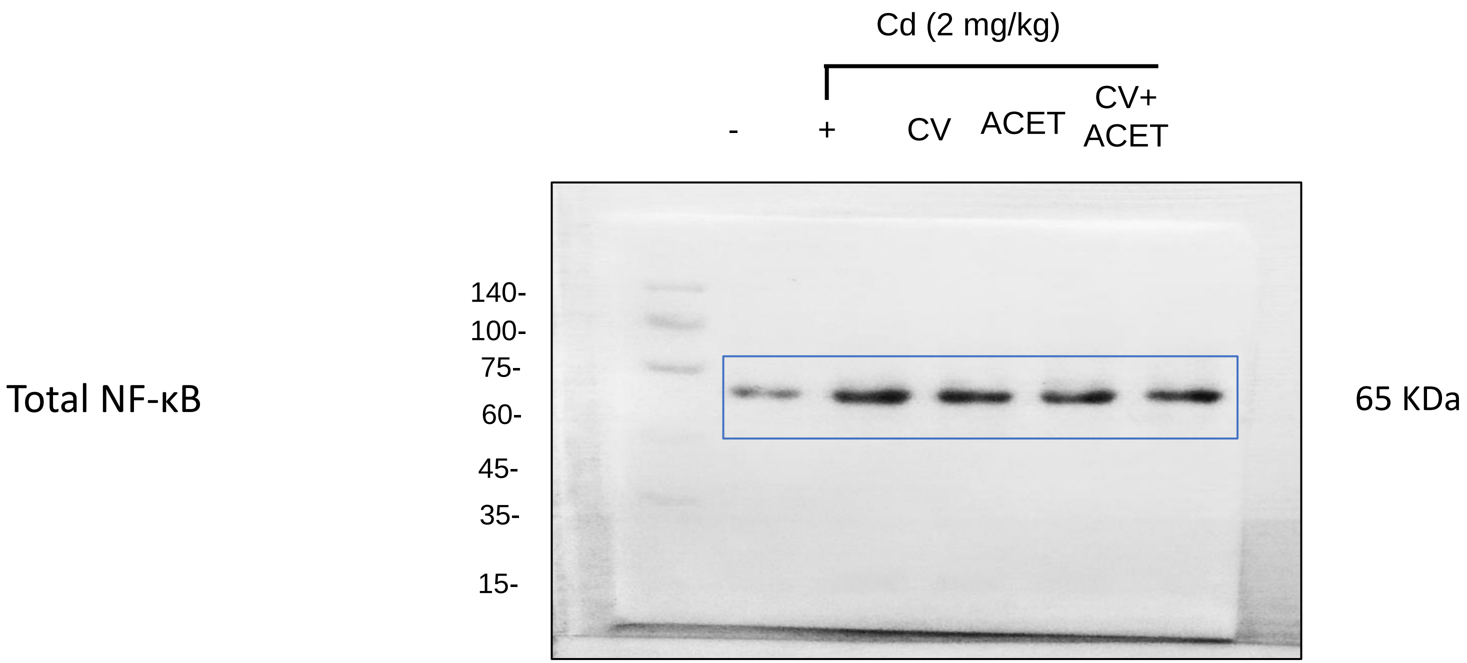
Cd (2 mg/kg)
-
+
CV
ACET
CV+ ACET
140-
100-
75-
60-
45-
35-
15-
Total NF-κB
65 KDa

β-actin

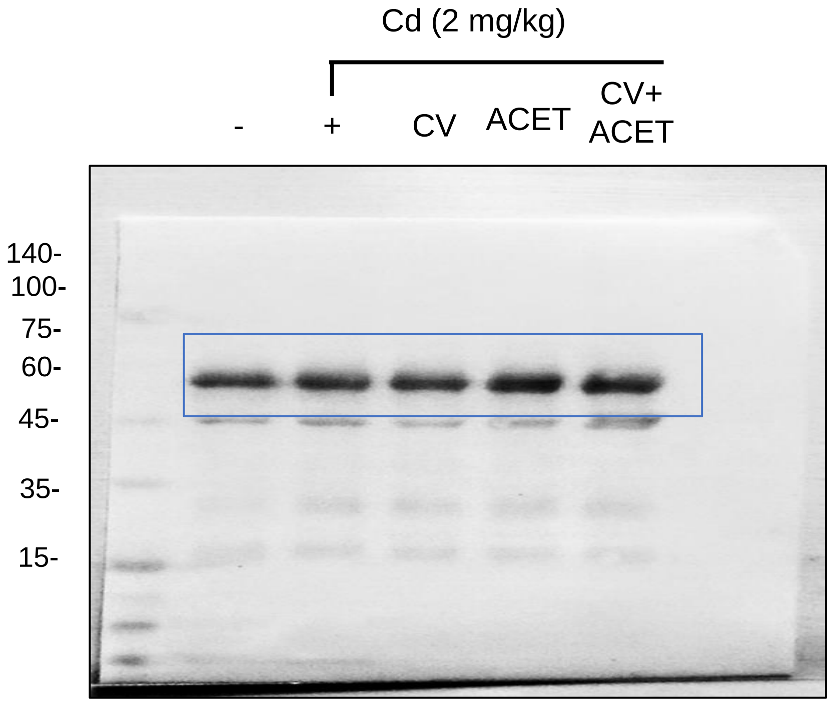

43 KDa

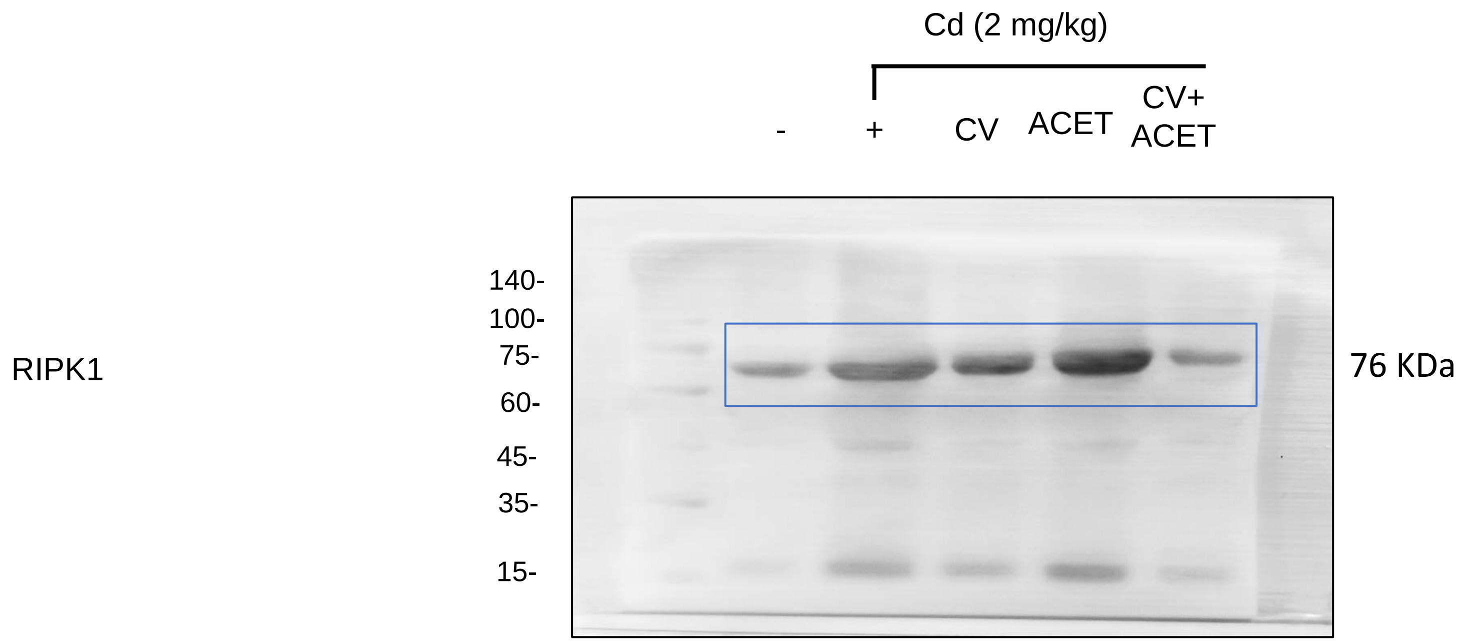
Cd (2 mg/kg)
-
+
CV
ACET
CV+ ACET
RIPK1
140-
100-
75-
60-
45-
35-
15-
76 KDa

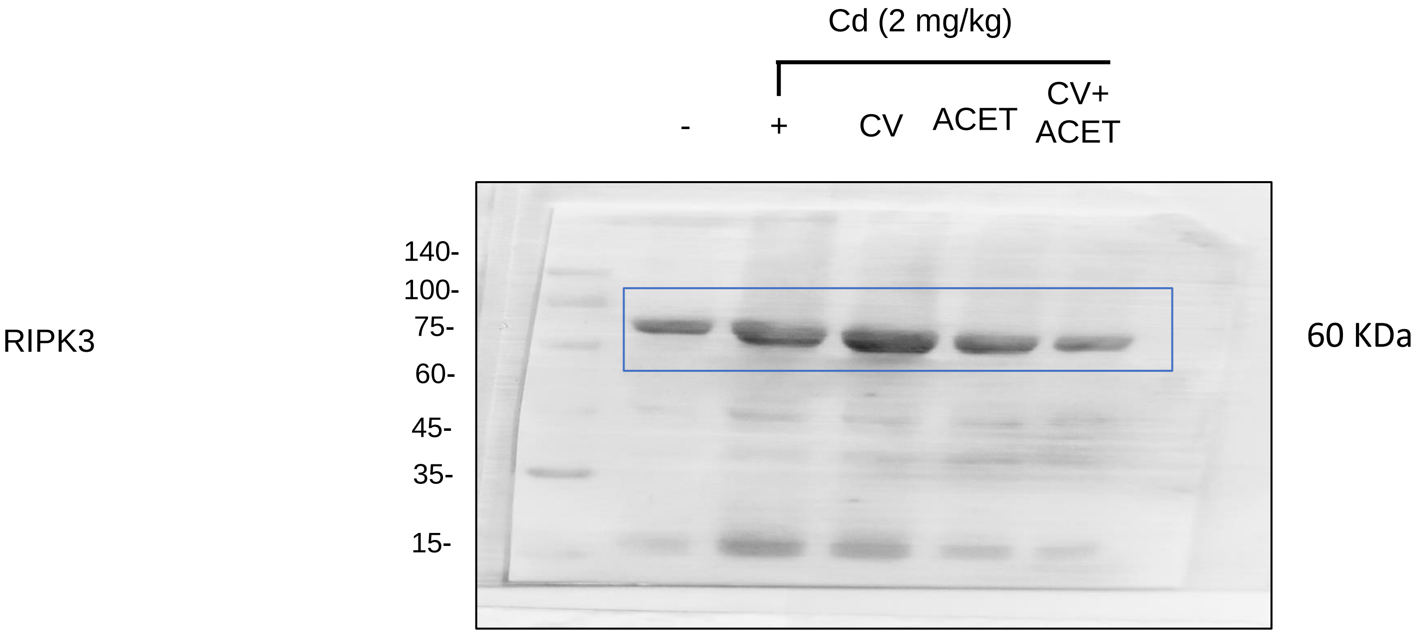
Cd (2 mg/kg)
-
+
CV
ACET
CV+
ACET
140-
100-
75-
60-
45-
35-
15-
RIPK3
60 KDa

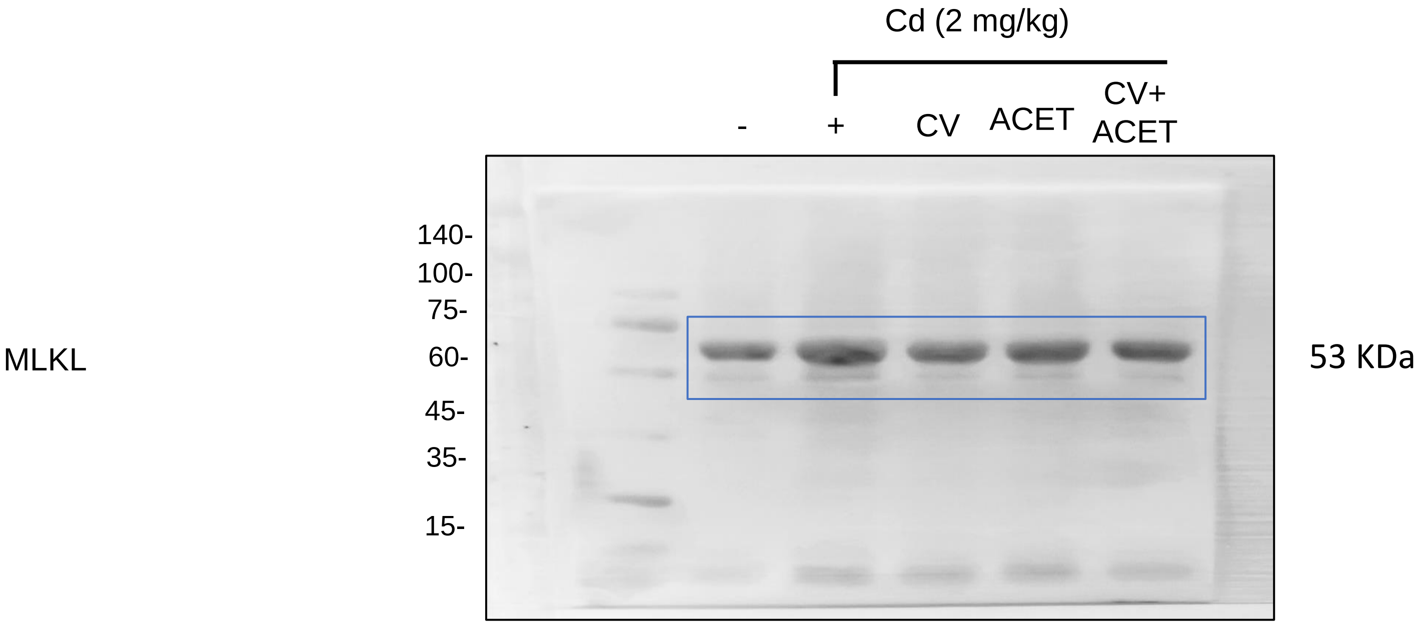

Cd (2 mg/kg)
-
+
CV
ACET
CV+
ACET
140-
100-
75-
60-
45-
35-
15-
MLKL
53 KDa

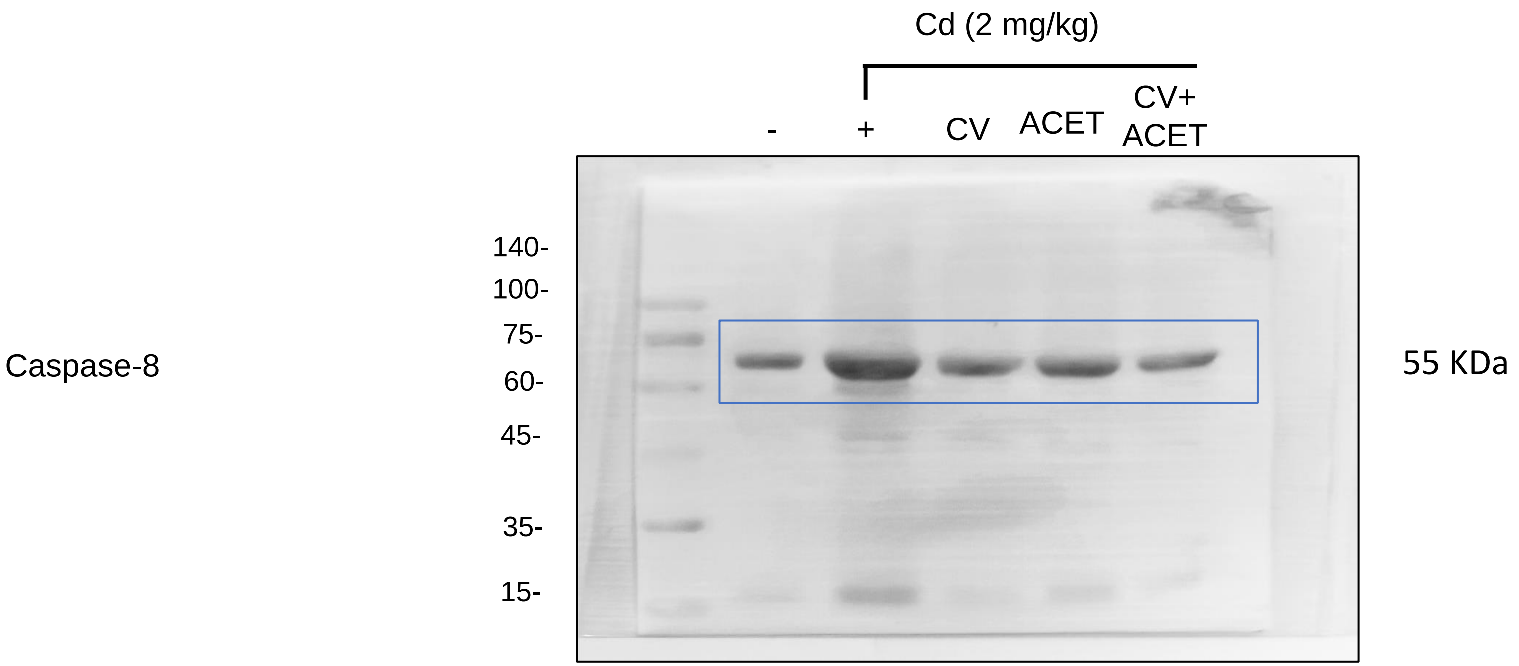
Cd (2 mg/kg)
-
+
CV
ACET
CV+
ACET
Caspase-8
140-
100-
75-
60-
45-
35-
15-
55 KDa

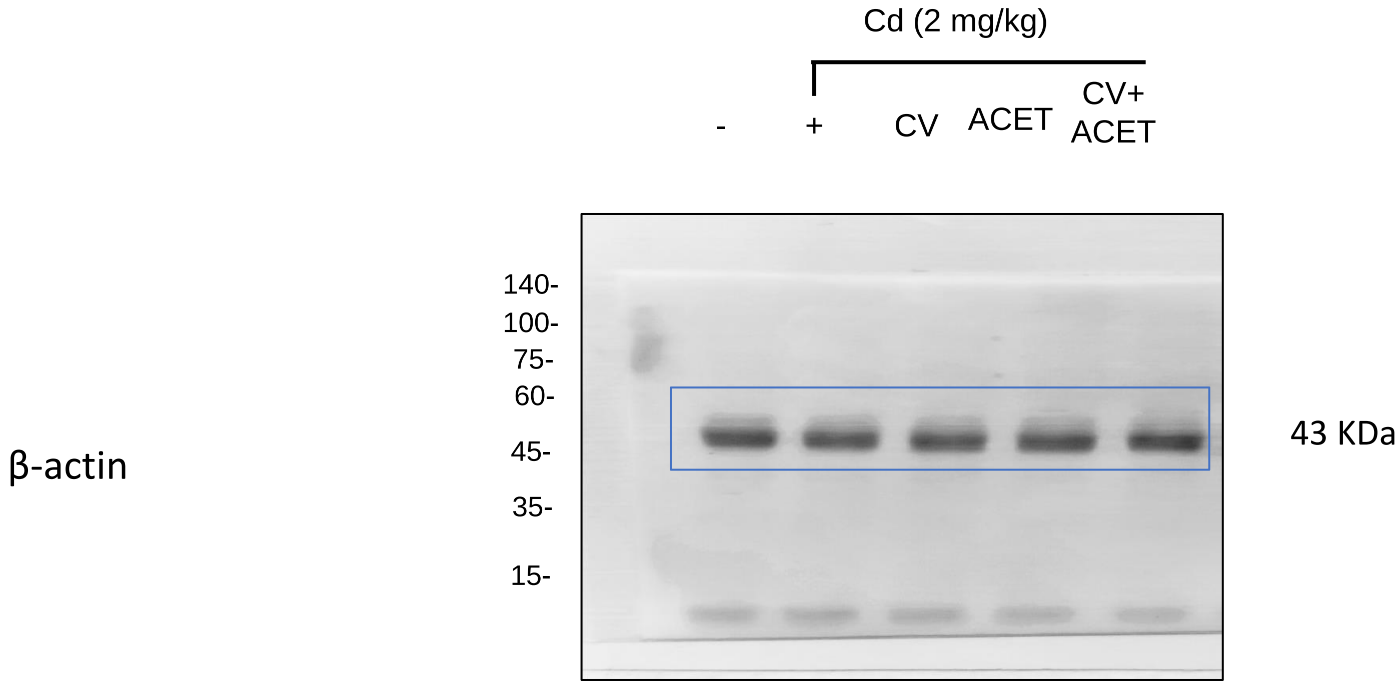
Cd (2 mg/kg)
-
+
CV
ACET
CV+ ACET
140-
100-
75-
60-
45-
35-
15-
β-actin
43 KDa
